# COVID-19 and persistent symptoms: implications for polycystic ovary syndrome and its management

**DOI:** 10.3389/fendo.2024.1434331

**Published:** 2024-10-04

**Authors:** Shanshan Zhang, Yanqun Wu, Richard Mprah, Mingming Wang

**Affiliations:** ^1^ School of Biological Science, Jining Medical University, Rizhao, Shandong, China; ^2^ Department of Physiology, School of Basic Medical Sciences, Xuzhou Medical University, Xuzhou, Jiangsu, China; ^3^ China National Experimental Teaching Demonstration Center for Basic Medicine, Xuzhou Medical University, Xuzhou, Jiangsu, China

**Keywords:** polycystic ovary syndrome, Long COVID, care strategies, treatment approaches, multidisciplinary management

## Abstract

The COVID-19 pandemic has left a profound mark on global health, leading to substantial morbidity and mortality worldwide. Beyond the immediate symptoms of infection, the emergence of “long COVID”, the long-term effects of SARS-CoV-2, has become a significant public health concern. Long COVID is a multifaceted condition affecting various organs and systems, including the cardiovascular, digestive, nervous, and endocrine systems. Individuals diagnosed with polycystic ovary syndrome (PCOS) may face an increased risk of severe COVID-19 symptoms and infection. It is crucial to comprehend how long COVID affects PCOS patients to devise effective treatment and care strategies. Here, we review the detrimental effects of COVID-19 and its long-term effects on reproductive health, endocrine function, inflammation, metabolism, cardiovascular health, body composition, lifestyle, and mental health in patients with PCOS. We offer recommendations for the post-covid-19 management of PCOS, emphasizing the necessity of a comprehensive, multidisciplinary approach to patient care. Furthermore, we discuss prospective research directions, highlighting the significance of continued investigations and clinical trials to evaluate treatment approaches for long COVID and its ramifications in individuals with PCOS.

## 1 Introduction

Coronavirus disease 2019 (COVID-19), caused by infection with severe acute respiratory syndrome coronavirus 2 (SAR-CoV-2), has been declared a public health emergency of international concern (1). SARS-CoV-2 enters cells by interacting with spike protein S and angiotensin-converting enzyme 2 (ACE2), causing organ dysfunction (2). The COVID-19 pandemic has taken a great toll worldwide, with profound consequences for individuals, organizations, and societies. While the initial focus was on the acute symptoms of the virus, more attention is now being paid to the long-term after-effects of COVID-19 (3).

**Figure 1:**
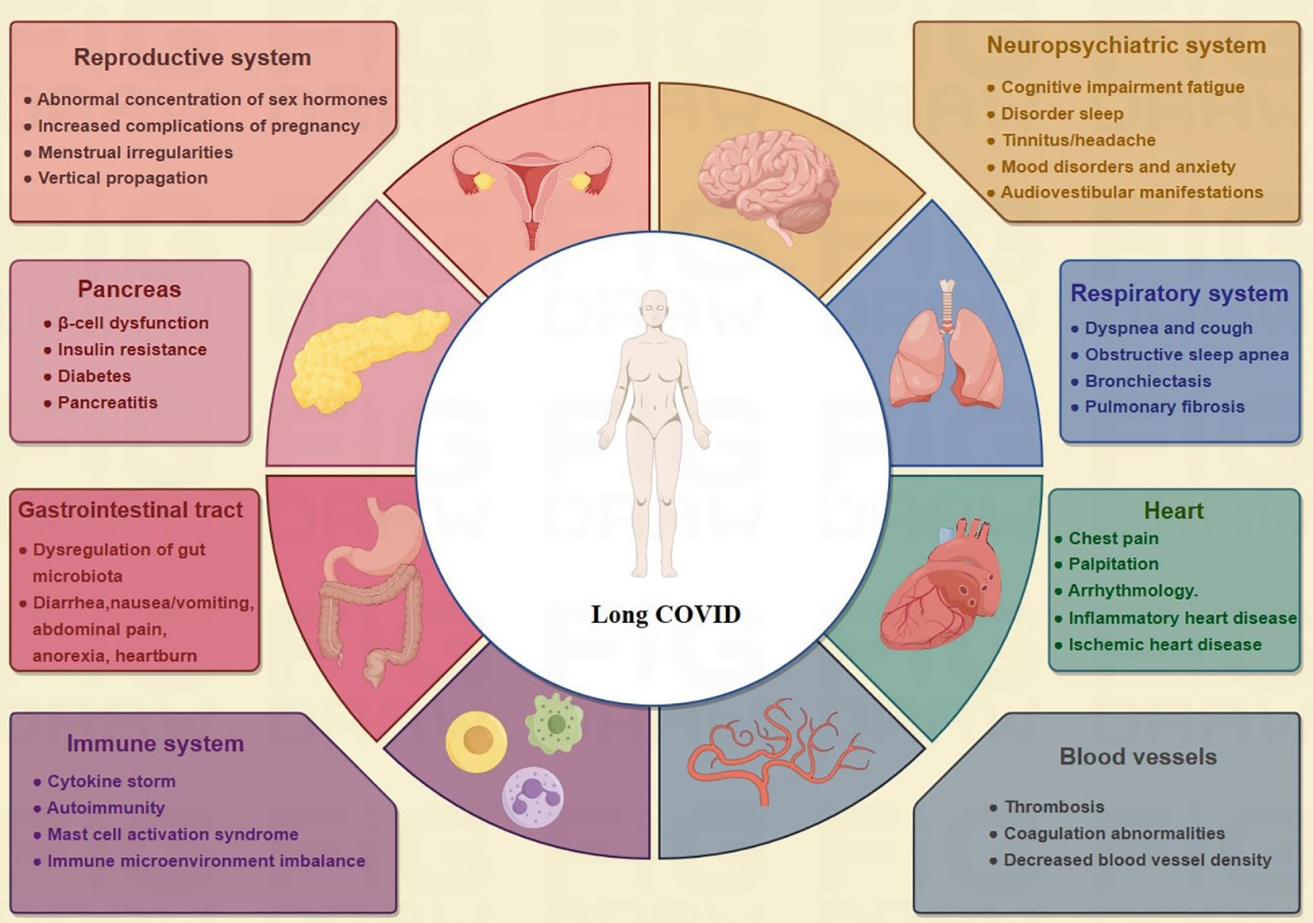
Multisystem symptoms/manifestations of long COVID. Among the various organ systems that long COVID can damage are the respiratory, cardiovascular, neuropsychological, digestive, circulatory, immune, and genitourinary systems, making it a truly multiorgan disease.

Even after the virus becomes undetectable in COVID-19 patients, it can continue to replicate for up to four weeks following infection, potentially resulting in long-term effects on various organs and systems. This condition is commonly referred to as ‘‘Long COVID” (4) (**Figure 1**). The Centers for Disease Control has listed approximately 25 clinical laboratory abnormalities associated with an increase in COVID-19 prevalence, which affects the health-related quality of life and well-being of COVID-19 patients (5, 6). It is estimated that 10-20% of cases across all ages, including children, will develop long COVID (a complex disorder of multiple organ system dysfunction), with most cases occurring in people with mild acute illness (7, 8).

**Figure 2:**
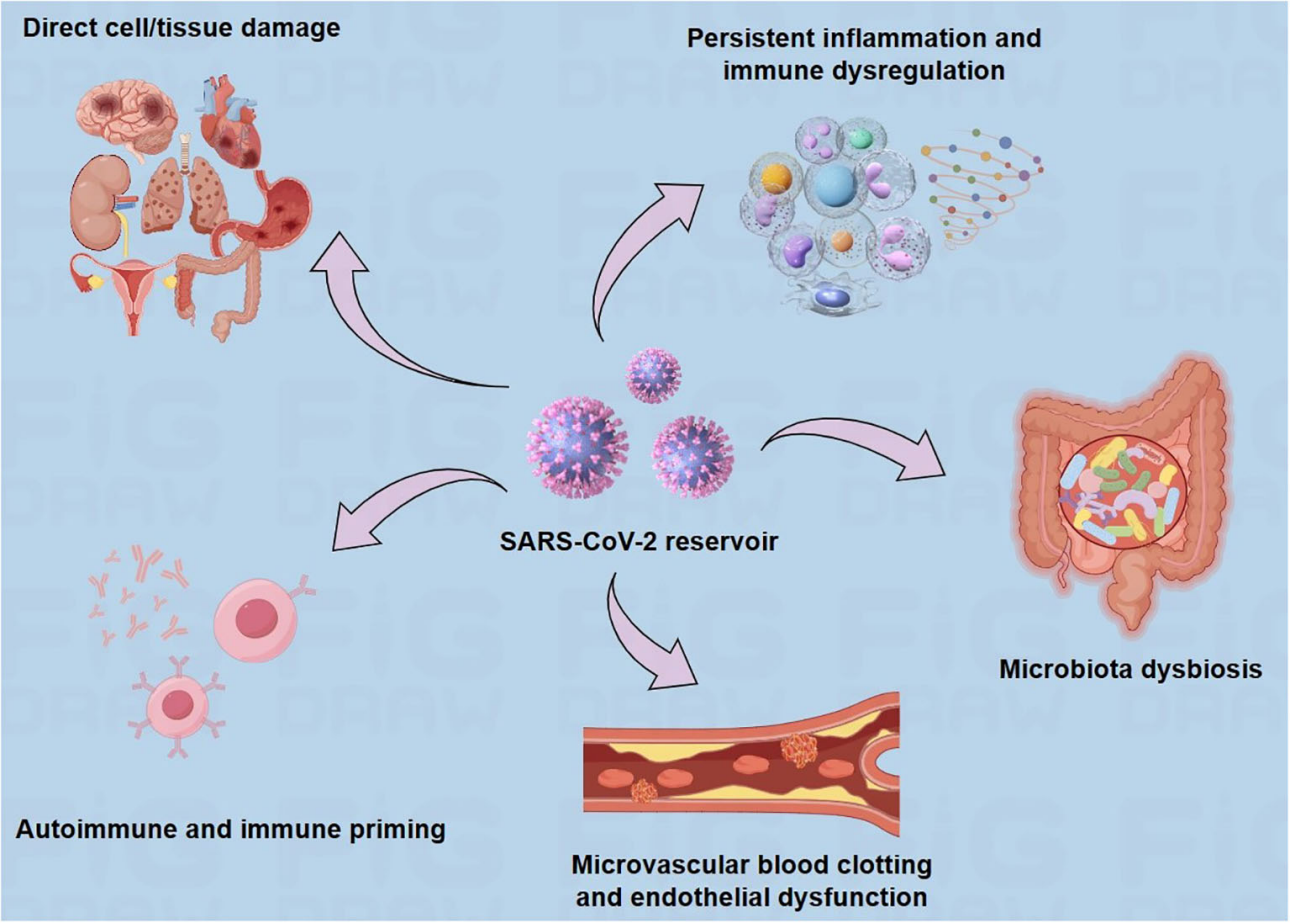
Suggested explanations for the underlying causes of long COVID. There are several hypothesized mechanisms for the pathogenesis of long COVID, including prolonged presence of the virus, direct cell/tissue damage, immune dysregulation, microbiome disruption, autoimmunity, coagulation, and endothelial abnormalities.

Several hypotheses for the pathogenesis of long COVID have been proposed (**Figure 2**), including the persistence of SARS-CoV-2 in tissues (9), pathological inflammation caused by persistent autoimmune responses and immune disorders (10, 11), long-term tissue damage (12), endothelial dysfunction and coagulation dysfunction (13), and the effects of SARS-CoV-2 on microbiota, including virome (10). Relevant risk factors may include female sex, type 2 diabetes, androgens, early dyspnoea, previous psychiatric disorders, and specific biomarkers (14). However, most of these studies on the mechanism hypothesis are preliminary, and further studies on the pathophysiology of long COVID are urgently needed.

**Figure 3:**
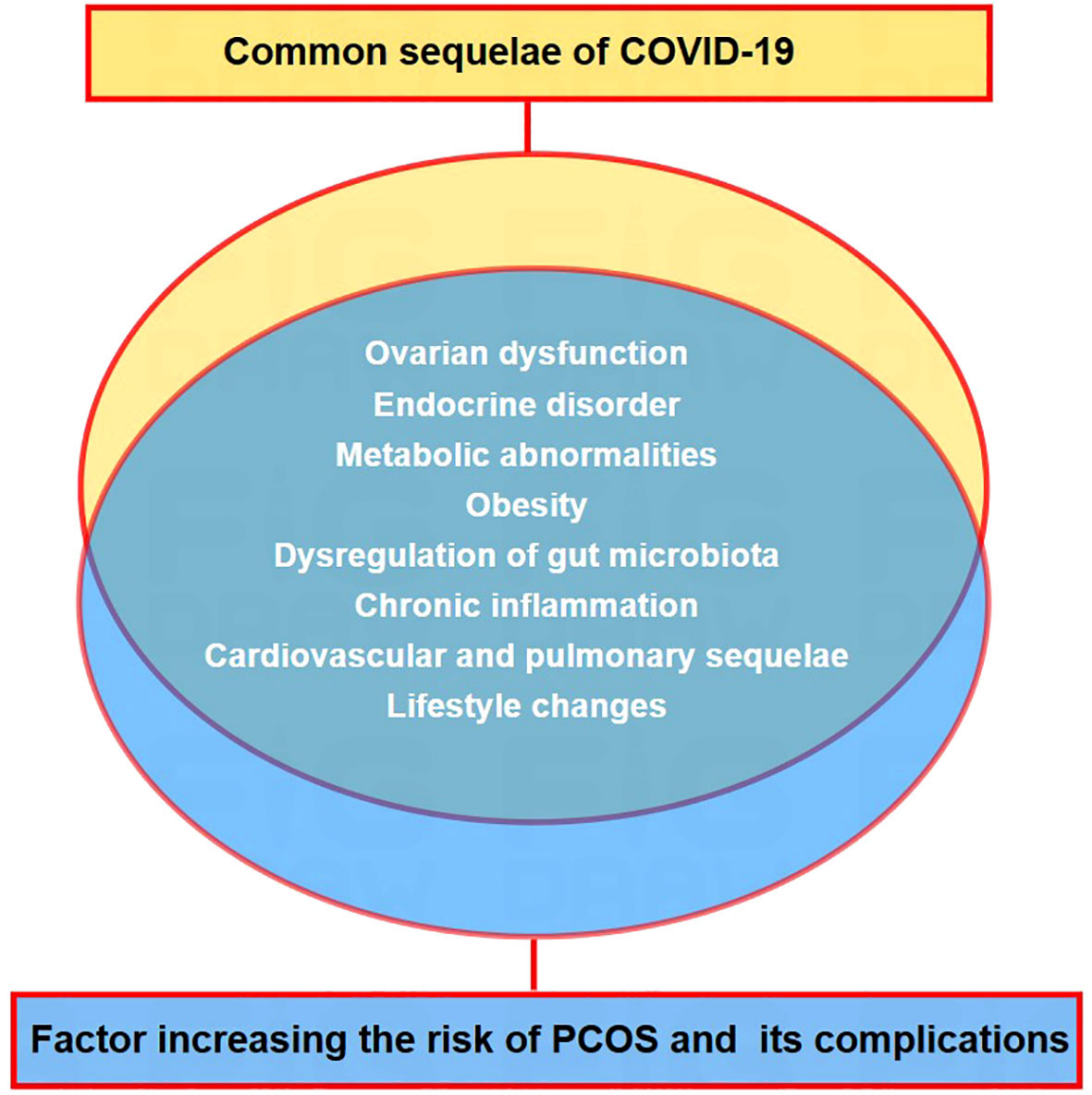
Convergence of post-covid-19 health issues and risk factors associated with PCOS and related problems. Overlapping factors between the common long-term outcomes of COVID-19 and risk indicators for PCOS and its associated health issues.

Polycystic ovary syndrome (PCOS) is an endocrine disorder affecting 5–20% of females of reproductive age (15). This syndrome can lead to infertility, insulin resistance (IR), obesity, type 2 diabetes, dyslipidaemia, cardiovascular problems, and a series of other health issues (16, 17). The overlap of many PCOS comorbidities with risk factors for severe COVID-19 progression has attracted research attention (**Figure 3**). Multiple studies have shown that women with PCOS are at a higher risk of contracting the SARS-CoV-2 virus and worsening COVID-19-related outcomes at all ages (18–20). A study of 21,000 patients with PCOS showed that women with PCOS had a 28% higher risk of developing COVID-19 (21). The clinical features of PCOS, such as hyperandrogenism, obesity, IR, chronic low-grade inflammation, and intestinal flora disturbance, may increase the risk of SARS-CoV-2 infection (18, 22). However, the impact of COVID-19 on patients with PCOS has not yet been explored. This review updates our knowledge on this issue, specifically examining the impact of COVID-19 and its subsequent effects on PCOS and its accompanying health issues.

To gain a comprehensive understanding of the health impact of COVID-19 and its long-term effects on patients with PCOS and to provide guidance for future research and clinical practice, we conducted an extensive literature search of reputable databases, including PubMed and Web of Science. The search terms involved “long-term COVID-19”, “polycystic ovary syndrome”, “epidemiology”, “symptoms”, “mechanism”, “management” and other related terms. Articles were selected based on their relevance to the topic identified in the abstract. The reference lists of articles were also searched to identify relevant literature.

## 2 Impacts of COVID-19 and its sequelae on PCOS

### 2.1 Reproductive health

#### 2.1.1 Menstrual irregularities

The female reproductive endocrine system is commonly known to be susceptible to various viruses. Evidence from studies demonstrates that COVID-19 patients underwent different levels of temporary menstrual changes, including longer menstrual cycles and decreased menstrual blood volume (23, 24). Following the COVID-19 pandemic, a study of 1,031 women found that 53% suffered from more severe premenstrual symptoms, 18% experienced new menorrhagia, and 30% experienced new dysmenorrhoea (25). One study revealed a link between menstrual disturbances and the gravity of COVID-19 (24). However, this change is temporary, and most patients return to normal within 1-2 months of discharge.

In addition to SARS-CoV-2 infection, the COVID-19 vaccine also affects the menstrual cycle (26). Studies have shown an increased incidence of changes in the menstrual cycle after COVID-19 vaccination, particularly in menstrual cycle length, menstrual pain, and flow of menstruation (27), which may be attributed to immunological processes (28, 29).

#### 2.1.2 Ovarian dysfunction

To date, no significant studies have reported the influence of COVID-19 on the ovarian reserve, function, or follicular fluid properties. However, clinical studies have shown that ovarian damage can be observed in women with COVID-19, including decreased ovarian reserve and reproductive endocrine disruption. Certain patients exhibit irregular changes in their sex hormone levels, including elevated follicle-stimulating, luteinizing hormones, testosterone, prolactin, and reduced estradiol and progesterone levels, potentially suggesting ovarian suppression (23, 30–32). Elevated levels of luteinizing hormones would stimulate theca cells to secrete more testosterone, potentially leading to secondary ovulation dysfunction at a later time. This situation could be even more problematic for women with PCOS who already have underlying endocrine disorders. Anti-Müllerian hormone (AMH) is secreted by small antral follicles and is an important indicator for evaluating the ovarian reserve. It is not affected by the menstrual cycle, exogenous sex hormones or pregnancy (33). Studies on the effects of COVID-19 on AMH have shown mixed results, with some studies showing no difference in average AMH concentrations in COVID-19 patients compared to controls (23, 34); however, others suggest that COVID-19 infection can lead to ovarian reserve impairment by reducing AMH levels (32, 35).

When the body experiences acute stress, ovarian function is typically suppressed to maintain normal functioning of vital organs, and cases of anovulation have been documented in numerous acute illnesses. SARS-CoV-2 has demonstrated the ability to invade host cells via either the ACE2/transmembrane serine protease 2 (TMPRSS2) pathway or the basigin/cathepsin L (BSG/CTSL) pathway (30). Public datasets have demonstrated that the coexistence of ACE2 and TMPRSS2 expression in ovaries is most prominent in oocytes, whereas a minor expression is also present in granulosa cells (36, 37). A comparison of ACE2-positive and ACE2-negative ovarian cells revealed greater enrichment of various viral infection-related pathways in the former (38), suggesting that SARS-CoV-2 specifically targets certain ovarian cells through the ACE2/TMPRSS2 pathway, thereby suppressing ovarian function.

### 2.2 Endocrine diseases

#### 2.2.1 The hypothalamic-pituitary-thyroid axis (HPT)

Thyroid dysfunction can exacerbate metabolic disorders, dyslipidaemia, cardiovascular disease risk, and reproductive health disorders in patients (39–41). Endocrine conditions, including thyroid dysfunction, adrenal dysfunction, and hyperandrogenism, have been linked to increased vulnerability to and severity of SARS-CoV-2 infection. Notably, coronavirus directly affects the thyroid gland (42). According to reports, COVID-19 inpatients may experience clinical thyroid dysfunction, such as thyrotoxicosis, hypothyroidism, and subclinical thyroid dysfunction, with the level of thyroid-stimulating hormone indicating the presence of hyperthyroidism or hypothyroidism (43). A study reported that 87% of patients continued to suffer from hypothyroidism even after more than three months following their recovery from COVID-19 (44). Currently, there is no evidence of a direct or indirect effect of SARS-CoV-2 on thyroid function. However, given that SARS-CoV-2 appears to be capable of causing organ damage through autoimmune processes (45), COVID-19-induced thyroid damage via immune system dysregulation cannot be ruled out.

#### 2.2.2 The hypothalamic–pituitary–adrenal axis (HPA)

HPA plays a crucial role in the female reproductive system, and adrenal cortex dysfunction often accompanies variations in reproductive system function (46). Multiple case studies and autopsy results have corroborated the deleterious effect of SARS-CoV-2 on the HPA axis (47–50). A study on the impact of SARS-CoV-2 on the HPA axis found that some COVID-19 patients had lower levels of dehydroepiandrosterone sulfate (DHEAS) and adrenocorticotropic hormone (ACTH) in the morning (51–53). COVID-19 patients may experience central adrenal insufficiency (54). COVID-19, through its cytopathic actions, might enhance the degradation and necrotic processes affecting adrenal cortical cells (55). Physiological stress caused by diseases, such as infection, trauma, surgery, sepsis, and critical illness, can activate the HPA axis, decrease cortisol metabolism and binding proteins, and increase serum cortisol levels (56). An increase in cortisol can trigger many neuroendocrine and immune adaptive adjustments within the body, ultimately resulting in stress responses (57). The pathological changes and physiological stress of the HPA caused by SARS-Cov2 may have negative effects on the reproductive system.

### 2.3 Metabolic abnormalities

#### 2.3.1 Obesity

Globally, the COVID-19 crisis has profoundly affected both physical and mental health (58). The implementation of COVID-19 restrictions and lockdowns has been associated with weight gain, with nearly 30% of the population experiencing this effect (59). Since the inception of the COVID-19 pandemic, there has been a staggering increase in obesity rates among children aged 2–17 (60). One study indicated that during the pandemic, pre-existing differences in obesity in terms of race, ethnicity, and community socioeconomic status have widened.

Increased SARS-CoV-2 replication enhances the inflammatory immune response, which leads to fat inflammation and IR in obesity (61). Recent evidence suggests that SARS-CoV-2 directly infects human adipocytes and alters cell metabolism in a depot-specific and viral lineage-dependent manner (62). SARS-CoV-2 infection inhibits lipolysis in subcutaneous adipocytes and increases pro-inflammatory gene expression in visceral adipocytes (63, 64). *In vitro* models suggest that viral infection directly alters the morphology and function of adipocytes (65).

Obesity increases IR and compensatory hyperinsulinaemia, leading to hyperandrogenemia, which in turn increases lipogenesis and decreases lipolysis (66). Obesity can interfere with ovarian function through neuroendocrine mechanisms, leading to ovulation disorders (67). Many bioactive molecules released by adipose tissue interact with multiple molecular pathways involved in IR, inflammation, hypertension, cardiovascular risk, coagulation, and oocyte differentiation and maturation, thereby amplifying and worsening the metabolic and reproductive phenotypes of PCOS (68). Obesity is also an important factor leading to the presence and severity of PCOS in adolescents, and intractable pre-adolescent obesity with severe IR may predict later development of PCOS (69, 70). It is plausible that obesity due to the COVID-19 pandemic may increase the prevalence of PCOS, particularly among adolescents.

#### 2.3.2 IR and β-cell dysfunction

PCOS is associated with severe IR and defective insulin secretion. IR and β-cell dysfunction are considered major drivers of PCOS pathophysiology and are involved in the occurrence of hyperandrogenemia and reproductive dysfunction through multiple mechanisms (71).

COVID-19 patients experience a cytokine storm, with a large number of inflammatory cells affecting the function of the skeletal muscle and liver, the two main insulin-responsive organs responsible for most insulin-mediated glucose uptake (72). Clinical studies have shown that severely ill patients with COVID-19 have a high demand for insulin during peak inflammatory responses, and this significant increase in insulin demand may be due to systemic inflammation and severe IR due to critical illness (73, 74). SARS-CoV-2 induces elevated cytokine levels that promote pancreatic β-cell over-stimulation and IR, resulting in fatigue and subsequent alterations in metabolism (75). COVID-19 can significantly shorten the life expectancy of people with type 2 diabetes (76, 77). It can also cause β-cell dysfunction, IR, and abnormal control of glucose metabolism in COVID-19 patients who have never been diagnosed with diabetes (78). Glucose abnormalities can last for at least 2 months after disease onset (79). It is expected that COVID-19 will further exacerbate IR in patients with PCOS. Montefuscono et al. reported hyperinsulinemia associated with COVID-19, suggesting that COVID-19 may lead to IR, which in turn leads to hyperglycemia (78). He et al. demonstrated that newly developed IR, rather than insulin deficiency, is the mechanism underlying hyperglycaemia after SARS-CoV-2 infection. Another study showed that COVID-19 increased the risk of IR in non-diabetic patients (80). Moreover, this IR condition persists even after the virus has cleared, meaning that COVID-19 patients may face long-term pathological effects. Taken together, the evidence suggests that COVID-19 exacerbates IR in patients with PCOS.

In reviewing the current literature, we found significant limitations and unexplored areas of research on the impact of COVID-19 on IR in PCOS patients. In particular, there have been few studies focusing on the specific effects of COVID-19 on PCOS patients, and few studies have explored its impact on IR, a core pathological process. Most studies have focused on the general clinical presentation of COVID-19, its induced complications, and how it affects a wide range of metabolic diseases, such as diabetes, but have overlooked the unique effects that may occur in this specific population of PCOS. In addition, the current understanding of how COVID-19 specifically acts on the mechanisms of IR in PCOS patients remains unclear. Although some studies have proposed that SARS-CoV-2 may affect insulin sensitivity by damaging islet beta cells and triggering cytokine storms, these hypothetical mechanisms need to be verified and refined by more empirical studies to build a more complete and accurate scientific picture.

### 2.4 Dysregulation of gut microbiota

Several pieces of scientific evidence strongly support that the microbiome significantly affects the aetiology and sustenance of PCOS, and changes in the intestinal flora may further aggravate metabolic disorders, cytokine storms, endocrine disorders, and hyperandrogenemia in women with PCOS (81, 82). In COVID-19 patients, the composition of the gut microbiota is altered, and in combination with inflammatory cytokines and blood indicators, it mirrors the gravity of illness and immune system dysfunction (83). ACE2 affects the expression of intestinal neutral amino acid transporters (84), thereby regulating the composition of the intestinal microbiota and regulating local and systemic immune responses (85). The ecological imbalance of intestinal flora lasted for up to 30 days after the resolution of the disease (79). The lingering presence of SARS-CoV-2 in the intestines of COVID-19 patients directly leads to the loss of the conjunction-dependent intestinal mucosal barrier in children with multisystem inflammatory syndrome (86), hinting at the possibility that the chronic presence of SARS-CoV-2 in the gastrointestinal tract can provoke alterations in the intestinal microbiome, resulting in long-term outcomes. Sustained changes in the fecal microbiome of COVID-19 patients have been observed, with many bacteria associated with more proinflammatory cytokines and increased disease complications. Given the profound influence of intestinal flora on the pathophysiology of PCOS and changes in intestinal flora in COVID-19 patients, it is reasonable to speculate that the changes in intestinal flora induced by COVID-19 may exacerbate the symptoms and comorbidities of PCOS patients. The potential synergies between the two diseases warrant further investigation, as COVID-19 may exacerbate pre-existing PCOS-related metabolic and endocrine disturbances through dysregulation of the gut microbiome.

However, the literature on this intersecting field remains limited and is filled with gaps. First, there is a lack of studies that specifically examine the effects of COVID-19-induced changes in the gut microbiota of PCOS patients. Most studies have focused on either PCOS or COVID-19 alone, failing to bridge the link between the two diseases through the gut microbiota. Second, the long-term effects of changes in gut microbiota associated with COVID-19 in patients with PCOS are largely unknown. Although some studies have shown that intestinal dysbiosis can persist in the early stages of the disease, its impact on PCOS symptoms, fertility outcomes, and overall quality of life remains to be clarified. In addition, the mechanism of potential interactions between COVID-19, the gut microbiota, and PCOS is unclear. Although ACE2 and its role in regulating gut microbiome composition and immune response provide a promising avenue for exploration, the specific pathways linking COVID-19, gut dysbiosis, and PCOS outcomes require further investigation.

### 2.5 Low-grade chronic inflammation

Pro-inflammatory cytokines play a pivotal role in the pathophysiology of PCOS, potentially underpinning many of its metabolic abnormalities. These cytokines have been linked to dysfunction and inflammation within adipose tissue (87), IR, and the pathophysiology of diabetes (88) while exerting a regulatory influence on ovarian function and hyperandrogenemia.

In patients with SARS-CoV-2 infection, there is a notable depletion in the absolute number and functional vigor of antiviral cytotoxic lymphocytes (89, 90), alongside severe impairment of specific T-cell subtypes (91). The apparent hyperactivity of the immune system induced by the virus coupled with concurrent bacterial infection can overwhelm its capacity, leading to a chronic inflammatory state with lasting adverse effects. Even after recovery, this persistent inflammatory cascade may manifest as a spectrum of chronic symptoms, including profound fatigue, dyspnoea, and joint discomfort, as well as psychological distress, such as anxiety and depression (79). The COVID-19 cytokine storm is characterized by rapid proliferation and hyperactivation of macrophages and natural killer cells and the overproduction of >150 inflammatory cytokines and chemical mediators released by immune or nonimmune cells (92). Mast cell activation syndrome and lymphopenia (i.e. B-cell and T-cell lymphocyte deficiencies) may be the cause of COVID-19 hyperinflammation and post-covid-19 illness (93, 94). Indeed, increased levels of pro-inflammatory markers (e.g. C-reactive protein, Interleukin-6, and D-dimer) and lymphopenia have been associated with long-term COVID (12).

It is plausible that the infection of SARS-CoV-2 in women with PCOS, who already harbor a background of low-grade inflammation, could further exacerbate this proinflammatory predisposition, thereby compounding various reproductive and metabolic dysfunctions.

### 2.6 Cardiopulmonary functional capacity

In terms of cardiopulmonary function, COVID-19 typically presents with cough and can precipitate a range of cardiovascular and pulmonary complications, such as diffuse alveolar injury and interstitial pulmonary fibrosis (95). Approximately 50% of patients continue to experience dyspnoea for months after recovery (96, 97). The pathophysiology of lung injury caused by SARS-CoV-2 includes its binding with ACE2 and cytokine storm (51). Decreased cardiorespiratory fitness and disrupted autonomic nervous system function, coupled with irregular heart rate recovery, could be contributing factors to the elevated cardiovascular risk observed in patients with PCOS (98). Concurrently, compromised lung function predisposes patients to glucose intolerance (51), IR (99), type 2 diabetes (100, 101), and cardiovascular diseases (102, 103). These adverse outcomes may stem from the direct impact of hypoxaemia on glucose and insulin regulation (104) as well as the inflammatory mediators and altered insulin signaling associated with pulmonary dysfunction (105, 106), potentially exacerbating the metabolic manifestations of PCOS.

### 2.7 Fertility outcomes

#### 2.7.1 Assisted reproduction technology (ART)

PCOS is a multifaceted endocrine disorder that often intersects with challenges related to fertility and pregnancy. For many women with PCOS, the path to successful conception often involves ART. Recent investigations into the impact of COVID-19 on *in vitro* fertilization (IVF) cycles have yielded noteworthy insights. Notably, while COVID-19 did not seem to compromise patients’ physical resilience or ovarian reserve during IVF procedures, there was a discernible decrease in the proportion of high-quality embryos (107). Furthermore, evidence suggests that while acute SARS-CoV-2 infection may not impede immediate ART outcomes, it could potentially exert adverse effects on oocyte production over the long term (i.e. beyond 180 days post-infection) (108). It has also been reported that the sperm concentration of couples was significantly reduced after exposure to COVID-19 (109). Hence, there should be at least three months (the time required for folliculogenesis and spermatogenesis) between a patient’s recovery from COVID-19 and the resumption of IVF treatment.

The onset of the COVID-19 pandemic has precipitated unforeseen disruptions across various spheres, including the delivery of non-emergency healthcare services. Notably, both the American Society for Reproductive Medicine and the European Society of Human Reproduction and Embryology independently recommend the temporary cessation of reproductive health care services (110). This suspension had profound repercussions for individuals and couples awaiting or undergoing fertility treatment. Delays resulting from the pandemic have forced some patients to face age-related limitations enforced by funding agencies, creating formidable barriers to treatment access, and prolonged waiting periods exacerbate anxiety, stress, and despondency among individuals grappling with infertility, consequently diminishing the success rates of ART (111, 112). A recent study revealed that postponing fertility treatment by a mere 12 months could lead to a substantial decrease in the likelihood of achieving a successful live birth through IVF among women aged 38-39 and 40-42, with percentages dropping by 18.8% and 22.4%, respectively (113). Furthermore, the economic and reproductive medicine response to the COVID-19 pandemic has reduced the affordability and accessibility of fertility care and has had a profound impact on IVF live birth rates (114–116).

#### 2.7.2 Pregnancy complications

Epidemiological research has indicated that women with PCOS are more susceptible to COVID-19 than those without (19, 117). Contracting SARS-CoV-2 during pregnancy increases the risk of complications such as spontaneous abortion, premature delivery, intrauterine growth restriction, and maternal renal failure or disseminated intravascular coagulation (118). Furthermore, there is an elevated risk of stillbirth (119). Several preexisting conditions and demographic factors, including chronic hypertension, preexisting diabetes, advanced maternal age, high body mass index, and non-white ethnicity, increase the likelihood of severe COVID-19 during pregnancy. Pregnant women with COVID-19 are more prone to preterm birth, heightened rates of caesarean birth (120), and potentially heightened risks of maternal mortality and ICU admission (121), with newborns being more frequently admitted to neonatal units. Pregnant women with PCOS may fall within the high-risk category for pregnancy-related complications.

#### 2.7.3 Vertical transmission

Vertical transmission, occurring when an infected pregnant woman passes the infection to her fetus or baby during pregnancy, delivery, or the postpartum period, is a significant concern. The transmission route can involve the placenta *in utero*, during delivery, or through breastfeeding during maternal-infant contact. ACE2, expressed in various maternal tissues including the placenta, human trophoblast ectoderm, fallopian tubes, ovaries, vagina, cervix, and endometrium, plays a role in this transmission process. Studies have reported a combined neonatal vertical transmission rate of SARS-CoV-2 at 3.2%, underscoring the potential for such transmission (122), with the severity of maternal illness linked to the likelihood of vertical foetal transmission (123).

Placental transmission likely serves as the primary mechanism of vertical transmission, with severe/critically ill mothers more prone to placental SARS-CoV-2 positivity (124). Another mechanism briefly considered is the cervicovaginal vertical transmission route, which involves exposure of the newborn to infected cells during delivery (125). However, most studies testing vaginal fluids from infected pregnant women yielded negative results for the virus (126). While breast milk from mothers infected with SARS-CoV-2 (the virus that causes COVID-19) may contain minimal amounts of viral RNA, evidence suggests that breastfeeding rarely leads to transmission of the virus to newborns (127). According to previous reports, 93% (68 out of 73) of infants born to mothers who tested positive for COVID-19 were asymptomatic. However, a small fraction of these infants experiences adverse effects, such as gastric bleeding, multiple organ failure, and, in some cases, mortality (120). Although foetal and neonatal mortality due to COVID-19 during pregnancy is rare, adverse neonatal morbidity may be associated with maternal infection, including respiratory diseases and hyperbilirubinemia, as reported by Norman et al. (128).

### 2.8 Lifestyle

#### 2.8.1 Physical activity

For patients with PCOS, managing weight is essential not only for symptom improvement and increasing the chances of pregnancy but also for overall health. However, owing to the challenging pathophysiology of PCOS, weight management is exceptionally difficult (129). The social distancing measures implemented during the COVID-19 pandemic, such as closures of social, educational, and recreational facilities, have resulted in behavioral changes that can negatively impact physical activity and promote sedentary behavior, potentially exacerbating chronic health conditions such as obesity (130, 131). The closure of sports and leisure facilities during the pandemic further disrupted weight management efforts in PCOS patients. Additionally, the lingering effects of COVID-19 on patients, including fatigue, respiratory issues, and joint pain, can hinder exercise routines and weight management (6, 132, 133).

#### 2.8.2 Sleep patterns

Sleep patterns have also been significantly affected during the pandemic, with many women with PCOS reporting negative effects on sleep quality (134). Insomnia and poor sleep health are common issues in women with PCOS and are associated with mental health problems, such as anxiety and depression, which exacerbate stress levels (135, 136). Sleep deprivation can also lead to the secretion of proinflammatory cytokines (137), metabolic changes, and disruption of appetite regulation (138–140). These factors are closely linked to the pathogenesis of PCOS.

#### 2.8.3 Dietary habits

Eating disorders and improper diets, especially those that are too low in plant protein and consume carbohydrates with a high glycaemic index, increase the risk of overweight adolescent girls with PCOS (141), causing inflammation, IR, and negatively affecting body composition. A diet lacking quality can lead to an imbalance in the microbiome, subsequently causing intestinal permeability and endotoxaemia. These conditions can exacerbate hyperinsulinaemia, resulting in elevated insulin levels. Such high insulin levels stimulate increased androgen production within ovaries, disrupting the normal follicular development process. This, in turn, worsens the clinical severity of PCOS (142). Additionally, dietary and environmental factors play a pivotal role in the developmental programming of PCOS female susceptibility gene variants (143).

The stress and shopping restrictions caused by isolation lead to changes in people’s eating habits, with a significant increase in sweet preference and frequency of eating (144). Stress causes subjects to overeat and consume super palatable convenience foods high in sugar and/or fat (145), replacing more nutrient-rich foods, and thus reducing dietary protein intake (146, 147). These foods can boost serotonin production, which has a positive effect on mood (148). However, it is associated with an increased risk of obesity, chronic inflammation, metabolic abnormalities, and cardiovascular disease (149), which have been shown to increase the risk of more serious complications from COVID-19 (150). Before the COVID-19 outbreak, the risk of eating disorders was more than four times higher in women with PCOS than in the control group (151). Given that people with PCOS are more prone to uncontrolled and emotional eating, the risk of eating disorders may be further elevated during the pandemic.

### 2.9 Healthcare systems

Despite the widespread occurrence of PCOS, its fundamental causes and biological mechanisms remain unclear. The approach to managing this condition in routine clinical settings is fragmented, with inconsistencies in the care provided by specialists such as general practitioners, endocrinologists, and gynaecologists (152). Furthermore, significant knowledge deficits persist among medical professionals concerning the diagnosis, treatment strategies, and comprehensive nature of PCOS manifestations (153–155). Previous studies have shown that PCOS patients are dissatisfied with the diagnosis and treatment they receive, and feel the need to seek specialist care for their condition (156). As a result, women with PCOS are often caught in the gap between relevant healthcare services, even without the enormous pressure that the COVID-19 pandemic is putting on clinical practice. Women with PCOS lack sufficient knowledge and access to contemporary healthcare services, a problem that has become even more apparent during the COVID-19 crisis (117).

The COVID-19 pandemic has placed enormous pressure on healthcare services, requiring the reorganization and reprioritization of resources and changes in healthcare delivery models. During the pandemic, PCOS patients face numerous challenges in accessing healthcare support, particularly when it comes to primary care physicians who serve as a crucial source of assistance (157–159). Contacting these professionals or scheduling face-to-face appointments is very challenging, compounding the stress and anxiety associated with PCOS (160–162). The limited availability of primary care, coupled with the suspension of specialized PCOS-related medical services and the uncertainty surrounding their resumption, creates a significant burden for individuals with PCOS, further exacerbating their condition and overall well-being (163, 164). Thus, the role of healthcare workers during the peak of the COVID-19 pandemic cannot be underestimated. The COVID-19 pandemic puts healthcare workers at unprecedented risk and under increased stress due to work (5). Admittedly, the incidence of COVID-19 sequelae among healthcare workers affects their long-term performance, negatively affecting the healthcare environment.

The impact of COVID-19 on access to healthcare and PCOS management varies widely across countries, mainly because of differences in healthcare systems, resources, and pandemic response strategies. Developed countries with strong healthcare systems are generally better able to cope with the surge in COVID-19 cases; however, routine healthcare services have been disrupted even in these countries (165, 166). Telemedicine plays a vital role in maintaining continuity of care, enabling patients to access consultations, laboratory tests, and prescriptions without having to physically visit a healthcare facility (167, 168). Developing countries with weaker healthcare systems face tougher challenges, many of which struggle to provide basic COVID-19 care, let alone maintain services for chronic diseases such as PCOS (169–172). In resource-constrained settings, healthcare providers must prioritize COVID-19 cases over other diseases, and shortages of essential medicines needed for PCOS management are more common in developing countries, further exacerbating the problem for PCOS patients (171–174).

### 2.10 Psychological impacts

The mental health of women with PCOS is a critical concern, as studies have shown that they are more susceptible to depression, anxiety, and stress than are women without non-PCOS women (175). Recent international guidelines emphasize that women with PCOS are at a higher risk of developing mental health issues such as depression and anxiety, especially during the pandemic. The pandemic has exacerbated the emotional burden experienced by women with PCOS, leading to increased psychological distress (175).

The COVID-19 crisis has not only impacted the physical health of patients with PCOS but has also taken a toll on their mental well-being. Many individuals with PCOS have reported worsening mental health, including feelings of low mood, anxiety, and depression (164). The pandemic has also raised concerns regarding the potential impact of PCOS on the risk of severe COVID-19, causing heightened health anxiety among patients. During this period, it was discovered that conditions commonly associated with PCOS, such as obesity and diabetes, carry an increased risk of severe illness and mortality due to COVID-19. This uncertainty regarding the influence of PCOS on COVID-19 risk has led to significant health anxiety and depression among many patients.

Limited access to primary care and suspension of specialized medical services related to PCOS during the pandemic made patients feel neglected, exacerbating their distress and anxiety (164). The suspension of fertility services caused severe anxiety, psychological stress, and a sense of isolation in some individuals struggling with PCOS (176).

Due to factors such as changes in sleep patterns, mandatory quarantine measures, and socioeconomic impacts, it is anticipated that global efforts to combat COVID-19 will have a negative effect on the mental well-being of the general population. However, individuals with PCOS who are already vulnerable may face potentially greater consequences for their mental health.

The impact of COVID-19 on the mental health of women with PCOS may vary depending on several factors. Women with PCOS often require regular medical follow-ups and symptom management. In countries where access to medical services is limited due to the outbreak, these women may face challenges in managing their condition, leading to increased anxiety and stress. In some countries, rapid response and efficient operation of health services have been achieved through the optimization of diagnosis and treatment processes and the introduction of intelligent management systems, and strong healthcare systems and telemedicine services may alleviate some of these challenges, thereby providing continuity of care and support during the pandemic (177, 178). Conversely, in countries with inefficient health services, patients may have to wait for long periods to access treatment, which not only exacerbates their physical suffering but can also trigger or worsen psychological problems (179–181). In countries with financial worries and limited or disrupted social support due to the pandemic, these women may feel more isolated and experience higher levels of anxiety and depression (182–184). In addition, the severity of the epidemic, awareness of PCOS, cultural attitudes, access to mental health services, and other factors in different countries will also have different impacts on the mental health of women with PCOS (185–187).

## 3 Interventions and management during the COVID-19 pandemic

### 3.1 Telemedicine and virtual care

The COVID-19 pandemic has overwhelmed healthcare systems worldwide, dramatically reducing or even eliminating the hospitalizations of individuals with other illnesses, particularly chronic conditions. Undoubtedly, the pandemic has changed the management of chronic diseases, such as PCOS, as well as the daily interactions between patients and healthcare providers. Telemedicine has been widely implemented during this period to effectively manage several chronic noncommunicable diseases, including arterial hypertension (188). Considerable evidence shows that telemedicine is an effective, safe, and satisfactory clinical option for day-to-day management of chronic diseases (114).

One of the most widely adopted areas of telemedicine is outpatient consultation or virtual clinics. Virtual consultations can avoid transportation to hospitals and unnecessary contact with waiting rooms, enabling continuity of care without exposing patients or healthcare professionals to potential risks. Virtual clinics can also prevent unnecessary visits, thereby reducing the number of people in emergency departments and improving the efficiency of medical resource allocation. Clinical practice reports indicate that the consultation rate of virtual clinics is 60–95% of that of regular clinics, and various chronic diseases are satisfactorily controlled through virtual care (189, 190).

Since lifestyle changes are the first-line treatment for PCOS, most patients struggle with adherence to lifestyle management, and mobile services that provide education on lifestyle modifications can be beneficial in treating PCOS. Mobile health apps have been shown to improve body weight and oocyte quality in PCOS patients (191). This reflects the effectiveness of mobile apps in facilitating lifestyle changes in PCOS patients. Telepsychotherapy offers an important additional treatment option for PCOS patients experiencing anxiety and depression during the COVID-19 pandemic. Another study confirmed that mobile health apps based on cross-theoretical models can reduce BMI, anxiety, and depression in PCOS patients and improve exercise and dietary adherence in these individuals in the long term (192).

### 3.2 Adjustments in reproductive care guidelines

Patients with PCOS have a higher incidence of pregnancy complications, such as preterm labor, caesarean section, miscarriage, gestational diabetes mellitus, gestational hypertension, and preeclampsia. Anatomical, physiological, and immunological changes during pregnancy may lead to a higher risk of severe SARS-CoV-2 infection in pregnant women (193). To address these concerns and prevent potential complications, various fertility societies globally, including the American Society of Reproductive Medicine, Canadian Society of Fertility and Andrology, European Society of Human Reproduction and Embryology, and International Federation of Fertility Societies, issued guidelines during the pandemic (194). These guidelines emphasize the importance of implementing mitigation measures and infection control protocols in fertility care units. Most of these guidelines recommend a temporary halt for new fertility treatments, including ovulation induction, intrauterine insemination, *in vitro* fertilization, and non-urgent gamete cryopreservation. They also suggested postponing embryo transfers, elective surgeries, and non-emergency diagnostic procedures. A COVID-19 Task Force was established to monitor the situation and provide updated guidance in alignment with local health authorities. Enhanced monitoring of pregnant women with PCOS, particularly those with comorbid conditions, such as hypertension and diabetes, is recommended during prenatal and perinatal care (195).

### 3.3 Patient education and support

PCOS is a prevalent and intricate disorder linked to metabolic syndrome, obesity, eating disorders, depression, and sleep apnea (196, 197). Prior to the COVID-19 pandemic, a survey revealed that women with PCOS felt that the existing information, resources, and education did not adequately address their needs. They expressed dissatisfaction with early diagnostic care and believed that clinicians lacked sufficient knowledge of their conditions (198, 199). There are evident knowledge gaps and discrepancies among residents and physicians in the diagnosis and treatment of PCOS (154, 155).

Epidemiological studies have shown that women with PCOS are more likely to be infected with SARS-CoV-2 than are women without PCOS (19). Risk factors for severe COVID-19 highly overlap with the common features of PCOS, and COVID-19 may have an impact on all aspects of PCOS care. Additionally, the accompanying hormonal disorders (such as IR or hyperandrogenemia) associated with COVID-19 may further complicate the clinical features of PCOS (19, 117). The elevated risk of COVID-19 poses challenges in accessing timely healthcare for patients with PCOS, making diagnosis and treatment more difficult.

Effective management of PCOS is crucial during the COVID-19 pandemic, necessitating closer monitoring and revision of care plans for this patient population. The challenges posed by the pandemic highlight the need for improved education, resources, and knowledge dissemination among healthcare professionals to ensure optimal care for patients with PCOS.

## 4 Impact of variants

COVID-19 and its evolving variants have demonstrated a significant impact on human health, especially in those with PCOS, which may in different ways exacerbate existing comorbidities in patients and introduce new health challenges.

Different variants of SARS-CoV-2 have been found to differ significantly in causing COVID-19-related symptoms and in severity (200, 201). Primordial strain infections are associated with a higher proportion of long-COVID symptoms and face a greater burden of disease and health costs than Alpha or Delta variants (202, 203).

Notably, some COVID-19 variants have shown increased transmissibility and virulence compared with the original SARS-CoV-2 strain (204, 205). This property could put people with PCOS - whose immune systems may already be compromised by comorbidities such as obesity, insulin resistance and chronic inflammation - at higher risk of infection.

In addition, SARS-CoV-2 mutants are not only more infectious than wild-type viruses but also have a particular tendency to infect obese individuals (206), which means that PCOS patients with symptoms of obesity need to be closely followed and actively treated.

Different variants can also trigger different immune responses, further affecting the balance of the immune system in PCOS patients (207). For example, certain variants may trigger intense autoantibody production or cytokine storms that exacerbate autoimmune symptoms associated with PCOS, such as hyperandrogenemia and chronic inflammation.

In summary, the impact of COVID-19 variants on PCOS patients is diverse, given their different manifestations of virulence, infectivity, and disruption to various physiological systems (208). This difference may directly lead to further deterioration of reproductive health, metabolic status, mental health, cardiovascular health, and immune system functions. Therefore, it is important to continuously monitor the potential risks of novel COVID-19 variants in PCOS patients and develop personalized management strategies.

## 5 Future perspectives

**Figure 4:**
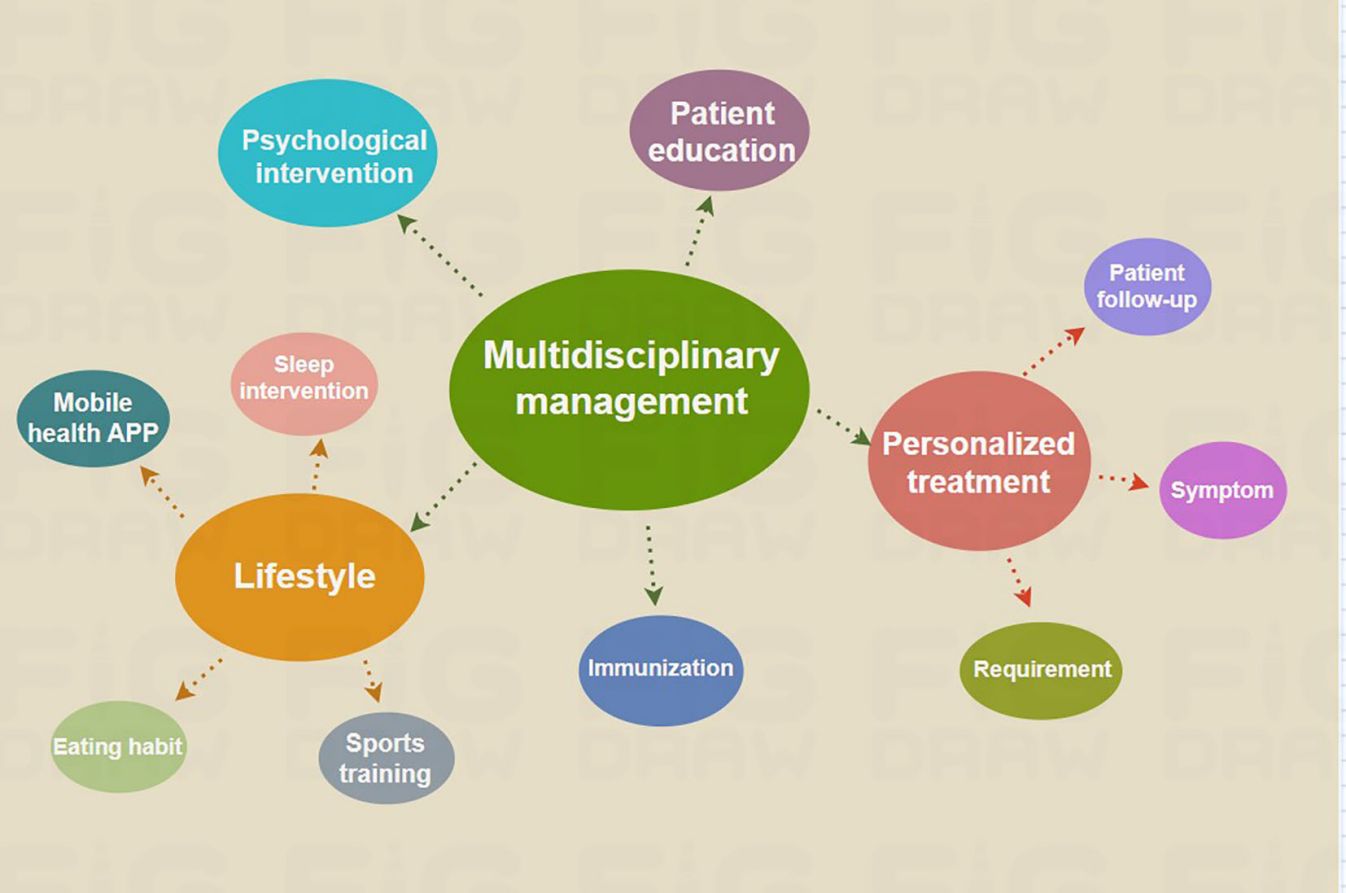
Therapeutic interventions for patients with PCOS affected by the sequelae of COVID-19. The complexity and wide-ranging impact of COVID-19 after-effects necessitate the use of a multidisciplinary care model for the optimal treatment of PCOS.

As the COVID-19 pandemic continues, its long-term impact, particularly on PCOS patients, highlights the urgency of a multifaceted approach to care (**Figure 4**). Long COVID exacerbates several challenges faced by women with PCOS, including immune, endocrine, metabolic, neurological, cardiovascular, and gastrointestinal issues. Recognizing this complexity, future research must delve into the mechanisms linking COVID-19 and PCOS to develop tailored interventions.

One of the crucial aspects of long-term COVID-19 management is the recognition and treatment of psychological issues, such as depression and anxiety, that can arise from the traumatic experience of the pandemic. Seeking counseling and psychological support is vital in helping survivors overcome feelings of despair and anxiety, and in turn, improve their overall quality of life.

Given the heterogeneity of PCOS, personalized treatment regimens are critical (121). Healthcare providers must consider the impact of long COVID on PCOS symptoms and adjust interventions accordingly. Involving people with PCOS in research and clinical trials will contribute to a deeper understanding of the disease and its interactions with COVID-19 (209).

The International Evidence-based Guidelines for the Evaluation and Management of PCOS emphasize the importance of lifestyle interventions, such as diet, exercise, and sleep optimization, for overweight or obese women with PCOS (210). These interventions are essential to improve the metabolic, hormonal, and psychological aspects of the syndrome. However, many women with PCOS may struggle to follow diet and exercise guidelines, especially in the post-pandemic world, where COVID-19 restrictions and concerns about disease transmission continue to affect physical activity levels. Mobile technology can improve compliance with lifestyle management recommendations by providing support and monitoring tools for PCOS patients.

Future research should explore drug treatments that target both PCOS symptoms and boost the immune system or target the virus itself (211). Leveraging current infrastructure, developing scalable healthcare models, and integrating them across disciplines (212) are essential to address the complex interplay between long COVID and PCOS. Long-term follow-up studies and the development of effective treatments are urgently needed, with a focus on the evolving phase of the pandemic, possible complications, vaccination status, and the presence of new viral strains (213).

Improving the diagnosis and treatment of PCOS remains a significant challenge in the healthcare field. Health professionals, especially primary care professionals, must be educated about the standard diagnostic criteria and treatment options. A multidisciplinary collaborative approach involving specialists in gynaecology, endocrinology, family medicine, psychiatry, and nutrition should be implemented. Continuing education programs should be developed for healthcare professionals and patients, including workshops, webinars, and standardized educational materials (214).

Subsequent research priorities should include (i) assessing the long-term effects of COVID-19 in people with or without PCOS; (ii) elucidating the causal mechanisms of COVID-19 and its sequelae affecting PCOS and its complications, including endocrine, immune system, metabolism, and mental stress; (iii) developing and improving scalable diagnostic methods that are highly specific for COVID-19-related PCOS complications; (iv) assessing the effects of vaccination and immunotherapy on PCOS and its complications; (v) identifying new therapeutic solutions or repurposing older drugs that can protect or reverse COVID-19-associated PCOS and its complications; and (vi) testing the feasibility and effectiveness of mobile health applications to improve health-related behaviors in women with PCOS.

In conclusion, a comprehensive and collaborative approach involving health care professionals, researchers, and patients is key to effectively managing the long-term effects of COVID-19 and PCOS. By prioritizing individualized care, psychological support, and ongoing research efforts, we can improve the outcomes and quality of life for those grappling with the aftermath of the pandemic. This approach should encompass lifestyle interventions, innovative technologies, and targeted drug therapies to address the unique challenges posed by the convergence of these conditions. By integrating these strategies, healthcare providers can better support patients in managing their health and well-being in the face of ongoing uncertainty and evolving healthcare needs.

## 6 Limitations

This review takes a multidisciplinary approach to provide patients with PCOS with a comprehensive perspective on understanding and managing the long-term effects of COVID-19. However, this method has several disadvantages. First, while the review cites a large number of studies, many of the studies on the relationship between long COVID and PCOS are still preliminary, leading to some conclusions that may not be deep or comprehensive enough. Second, the specific pathogenesis of long COVID is not fully understood at present, which limits the depth and accuracy of the review in exploring its mechanisms of influence. Finally, owing to the short duration of the COVID-19 outbreak, long-term follow-up data are relatively scarce, which has hindered the comprehensive assessment of the long-term impact of the long COVID.

## 7 Conclusion

The COVID-19 pandemic has significantly impacted global health, with long COVID emerging as a major concern. Long COVID affects multiple systems, posing a particular challenge for PCOS patients who may experience aggravated symptoms and complicated management. Current evidence indicates that COVID-19 and its sequelae negatively impact reproductive health, endocrine function, inflammation, metabolism, cardiorespiratory health, body composition, lifestyle, and mental health in patients with PCOS. These mechanisms are multifactorial and include inflammation, lifestyle changes, and comorbidities. Given the lack of effective therapies for PCOS post-COVID-19, a comprehensive multidisciplinary approach is crucial for its management. Future research and clinical trials are needed to evaluate treatment and prevention strategies, emphasizing the importance of personalized care and risk assessment in patients with PCOS and COVID-19.

## References

[1] Guan WJ Ni ZY Hu Y Liang WH Ou CQ He JX. Clinical characteristics of coronavirus disease 2019 in China. New Engl J Med. (2020) 382:1708–20. doi: 10.1056/NEJMoa2002032 PMC7092819 32109013

[2] Ni W Yang X Yang D Bao J Li R Xiao Y. Role of angiotensin-converting enzyme 2 (ACE2) in COVID-19. Crit Care (London England). (2020) 24:422. doi: 10.1186/s13054-020-03120-0 PMC7356137 32660650

[3] Parotto M Myatra SN Munblit D Elhazmi A Ranzani OT Herridge MS. Recovery after prolonged ICU treatment in patients with COVID-19. Lancet Respir Med. (2021) 9:812–4. doi: 10.1016/S2213-2600(21)00318-0 PMC8280055 34273267

[4] Szabo S Zayachkivska O Hussain A Muller V. What is really ‘Long COVID’? Inflammopharmacology. (2023) 31:551–7. doi: 10.1007/s10787-023-01194-0 PMC10039447 36964860

[5] Shukla AK Atal S Banerjee A Jhaj R Balakrishnan S Chugh PK. An observational multi-centric COVID-19 sequelae study among health care workers. Lancet regional Health Southeast Asia. (2023) 10:100129. doi: 10.1016/j.lansea.2022.100129 36531928 PMC9744681

[6] Xiong Q Xu M Li J Liu Y Zhang J Xu Y. Clinical sequelae in COVID-19 survivors in Wuhan, China: A single-centre longitudinal study. Clin Microbiol infection: Off Publ Eur Soc Clin Microbiol Infect Diseases. (2021) 27:89–95. doi: 10.1016/j.cmi.2020.09.023 PMC7510771 32979574

[7] The L. Long COVID: 3 years in. Lancet (London England). (2023) 401:795. doi: 10.1016/S0140-6736(23)00493-2 36906338 PMC9998094

[8] Lechner-Scott J Levy M Hawkes C Yeh A Giovannoni G. Long COVID or post-covid-19 syndrome. Multiple sclerosis related Disord. (2021) 55:103268. doi: 10.1016/j.msard.2021.103268 PMC8447548 34601388

[9] Swank Z Senussi Y Manickas-Hill Z Yu XG Li JZ Alter G. Persistent circulating severe acute respiratory syndrome coronavirus 2 spike is associated with post-acute coronavirus disease 2019 sequelae. Clin Infect diseases: an Off Publ Infect Dis Soc America. (2023) 76:e487–e90. doi: 10.1093/cid/ciac722 PMC10169416 36052466

[10] Proal AD VanElzakker MB. Long COVID or post-acute sequelae of COVID-19 (PASC): an overview of biological factors that may contribute to persistent symptoms. Front Microbiol. (2021) 12:698169. doi: 10.3389/fmicb.2021.698169 34248921 PMC8260991

[11] Phetsouphanh C Darley DR Wilson DB Howe A Munier CML Patel SK. Immunological dysfunction persists for 8 months following initial mild-to-moderate SARS-CoV-2 infection. Nat Immunol. (2022) 23:210–6. doi: 10.1038/s41590-021-01113-x 35027728

[12] Yong SJ. Long COVID or post-covid-19 syndrome: putative pathophysiology, risk factors, and treatments. Infect Dis (London England). (2021) 53:737–54. doi: 10.1080/23744235.2021.1924397 PMC8146298 34024217

[13] Osiaevi I Schulze A Evers G Harmening K Vink H Kümpers P. Persistent capillary rarefication in long COVID syndrome. Angiogenesis. (2023) 26:53–61. doi: 10.1007/s10456-022-09850-9 35951203 PMC9366128

[14] Davis HE McCorkell L Vogel JM Topol EJ. Long COVID: major findings, mechanisms and recommendations. Nat Rev Microbiol. (2023) 21:133–46. doi: 10.1038/s41579-022-00846-2 PMC9839201 36639608

[15] March WA Moore VM Willson KJ Phillips DI Norman RJ Davies MJ. The prevalence of polycystic ovary syndrome in a community sample assessed under contrasting diagnostic criteria. Hum Reprod (Oxford England). (2010) 25:544–51. doi: 10.1093/humrep/dep399 19910321

[16] Norman RJ Dewailly D Legro RS Hickey TE. Polycystic ovary syndrome. Lancet (London England). (2007) 370:685–97. doi: 10.1016/S0140-6736(07)61345-2 17720020

[17] Patel S. Polycystic ovary syndrome (PCOS), an inflammatory, systemic, lifestyle endocrinopathy. J Steroid Biochem Mol Biol. (2018) 182:27–36. doi: 10.1016/j.jsbmb.2018.04.008 29678491

[18] Morgante G Troìa L De Leo V. Coronavirus Disease 2019 (SARS-CoV-2) and polycystic ovarian disease: Is there a higher risk for these women? J Steroid Biochem Mol Biol. (2021) 205:105770. doi: 10.1016/j.jsbmb.2020.105770 33065278 PMC7550902

[19] Subramanian A Anand A Adderley NJ Okoth K Toulis KA Gokhale K. Increased COVID-19 infections in women with polycystic ovary syndrome: a population-based study. Eur J endocrinology. (2021) 184:637–45. doi: 10.1530/EJE-20-1163 PMC8052516 33635829

[20] Jerzak M Szafarowska M. Preliminary results for personalized therapy in pregnant women with polycystic ovary syndrome during the COVID-19 pandemic. Archivum immunologiae therapiae experimentalis. (2022) 70:13. doi: 10.1007/s00005-022-00650-z PMC8943102 35325391

[21] de Medeiros SF Yamamoto MMW de Medeiros MAS Yamamoto A Barbosa BB. Polycystic ovary syndrome and risks for COVID-19 infection: A comprehensive review: PCOS and COVID-19 relationship. Rev endocrine Metab Disord. (2022) 23:251–64. doi: 10.1007/s11154-022-09715-y PMC8881900 35218458

[22] Ilias I Goulas S Zabuliene L. Polycystic ovary syndrome: Pathways and mechanisms for possible increased susceptibility to COVID-19. World J Clin cases. (2021) 9:2711–20. doi: 10.12998/wjcc.v9.i12.2711 PMC8058679 33969054

[23] Li K Chen G Hou H Liao Q Chen J Bai H. Analysis of sex hormones and menstruation in COVID-19 women of child-bearing age. Reprod biomedicine online. (2021) 42:260–7. doi: 10.1016/j.rbmo.2020.09.020 PMC7522626 33288478

[24] Khan SM Shilen A Heslin KM Ishimwe P Allen AM Jacobs ET. SARS-CoV-2 infection and subsequent changes in the menstrual cycle among participants in the Arizona CoVHORT study. Am J obstetrics gynecology. (2022) 226:270–3. doi: 10.1016/j.ajog.2021.09.016 PMC8452349 34555320

[25] Phelan N Behan LA Owens L. The impact of the COVID-19 pandemic on women’s reproductive health. Front endocrinology. (2021) 12:642755. doi: 10.3389/fendo.2021.642755 PMC8030584 33841334

[26] Taşkaldıran I Vuraloğlu E Bozkuş Y Turhan İyidir Ö Nar A Başçıl Tütüncü N. Menstrual changes after COVID-19 infection and COVID-19 vaccination. Int J Clin practice. (2022) 2022:3199758. doi: 10.1155/2022/3199758 PMC9633189 36349056

[27] Aljehani AM Banjar SA Alawam HS Alowais S Aldraibi Y BinSaif A. The relationship between menstrual cycle irregularities and COVID-19 vaccination. Cureus. (2023) 15:e49841. doi: 10.7759/cureus.49841 38164312 PMC10758269

[28] Muharam R Agiananda F Budiman YF Harahap JS Prabowo KA Azyati M. Menstrual cycle changes and mental health states of women hospitalized due to COVID-19. PloS One. (2022) 17:e0270658. doi: 10.1371/journal.pone.0270658 35749547 PMC9231764

[29] Muhaidat N Alshrouf MA Azzam MI Karam AM Al-Nazer MW Al-Ani A. Menstrual symptoms after COVID-19 vaccine: A cross-sectional investigation in the MENA region. Int J women’s Health. (2022) 14:395–404. doi: 10.2147/IJWH.S352167 35378876 PMC8976114

[30] Ata B Vermeulen N Mocanu E Gianaroli L Lundin K Rautakallio-Hokkanen S. SARS-CoV-2, fertility and assisted reproduction. Hum Reprod update. (2023) 29:177–96. doi: 10.1093/humupd/dmac037 PMC9976972 36374645

[31] Wei L Sun S Zhang J Zhu H Xu Y Ma Q. Endocrine cells of the adenohypophysis in severe acute respiratory syndrome (SARS). Biochem Cell Biol = Biochimie biologie cellulaire. (2010) 88:723–30. doi: 10.1139/O10-022 20651845

[32] Ding T Wang T Zhang J Cui P Chen Z Zhou S. Analysis of ovarian injury associated with COVID-19 disease in reproductive-aged women in wuhan, China: an observational study. Front Med. (2021) 8:635255. doi: 10.3389/fmed.2021.635255 PMC8017139 33816526

[33] Iliodromiti S Anderson RA Nelson SM. Technical and performance characteristics of anti-Müllerian hormone and antral follicle count as biomarkers of ovarian response. Hum Reprod update. (2015) 21:698–710. doi: 10.1093/humupd/dmu062 25489055

[34] Madendag IC Madendag Y Ozdemir AT. COVID-19 disease does not cause ovarian injury in women of reproductive age: an observational before-and-after COVID-19 study. Reprod biomedicine online. (2022) 45:153–8. doi: 10.1016/j.rbmo.2022.03.002 PMC8897265 35523708

[35] Ghaemi M Hantoushzadeh S Shafiee A Gargari OK Fathi H Eshraghi N. The effect of COVID-19 and COVID-19 vaccination on serum anti-Mullerian hormone: A systematic review and meta-analysis. Immunity Inflammation disease. (2024) 12:e1136. doi: 10.1002/iid3.1136 38270314 PMC10777886

[36] Qi J Zhou Y Hua J Zhang L Bian J Liu B. The scRNA-seq expression profiling of the receptor ACE2 and the cellular protease TMPRSS2 reveals human organs susceptible to SARS-coV-2 infection. Int J Environ Res Public Health. (2021) 18(1):284. doi: 10.3390/ijerph18010284 33401657 PMC7794913

[37] Hikmet F Méar L Edvinsson Å Micke P Uhlén M Lindskog C. The protein expression profile of ACE2 in human tissues. Mol Syst Biol. (2020) 16:e9610. doi: 10.15252/msb.20209610 32715618 PMC7383091

[38] Wu M Ma L Xue L Zhu Q Zhou S Dai J. Co-expression of the SARS-CoV-2 entry molecules ACE2 and TMPRSS2 in human ovaries: Identification of cell types and trends with age. Genomics. (2021) 113:3449–60. doi: 10.1016/j.ygeno.2021.08.012 PMC8372464 34418496

[39] Fan H Ren Q Sheng Z Deng G Li L. The role of the thyroid in polycystic ovary syndrome. Front endocrinology. (2023) 14:1242050. doi: 10.3389/fendo.2023.1242050 PMC10585146 37867519

[40] Gaberšček S Zaletel K Schwetz V Pieber T Obermayer-Pietsch B Lerchbaum E. Mechanisms in endocrinology: thyroid and polycystic ovary syndrome. Eur J endocrinology. (2015) 172:R9–21. doi: 10.1530/EJE-14-0295 25422352

[41] Trouva A Alvarsson M Calissendorff J Åsvold BO Vanky E Hirschberg AL. Thyroid status during pregnancy in women with polycystic ovary syndrome and the effect of metformin. Front endocrinology. (2022) 13:772801. doi: 10.3389/fendo.2022.772801 PMC8898827 35265033

[42] Scappaticcio L Pitoia F Esposito K Piccardo A Trimboli P. Impact of COVID-19 on the thyroid gland: an update. Rev endocrine Metab Disord. (2021) 22:803–15. doi: 10.1007/s11154-020-09615-z PMC7688298 33241508

[43] Pal R Banerjee M. COVID-19 and the endocrine system: exploring the unexplored. J endocrinological Invest. (2020) 43:1027–31. doi: 10.1007/s40618-020-01276-8 PMC7195612 32361826

[44] Brancatella A Viola N Rutigliano G Sgrò D Santini F Latrofa F. Subacute thyroiditis during the SARS-coV-2 pandemic. J Endocrine Soc. (2021) 5:bvab130. doi: 10.1210/jendso/bvab130 34458656 PMC8344892

[45] Lyons-Weiler J. Pathogenic priming likely contributes to serious and critical illness and mortality in COVID-19 via autoimmunity. J Trans autoimmunity. (2020) 3:100051. doi: 10.1016/j.jtauto.2020.100051 PMC7142689 32292901

[46] Wang F Zhang ZH Xiao KZ Wang ZC. Roles of hypothalamic-pituitary-adrenal axis and hypothalamus-pituitary-ovary axis in the abnormal endocrine functions in patients with polycystic ovary syndrome. Zhongguo yi xue ke xue yuan xue bao Acta Academiae Medicinae Sinicae. (2017) 39:699–704. doi: 10.3881/j.issn.1000-503X.2017.05.017 29125115

[47] Hashim M Athar S Gaba WH. New onset adrenal insufficiency in a patient with COVID-19. BMJ Case Rep. (2021) 14:e237690. doi: 10.1136/bcr-2020-237690 PMC7813399 33462013

[48] Sheikh AB Javaid MA Sheikh AAE Shekhar R. Central adrenal insufficiency and diabetes insipidus as potential endocrine manifestations of COVID-19 infection: a case report. Pan Afr Med J. (2021) 38:222. doi: 10.11604/pamj.2021.38.222.28243 34046127 PMC8140727

[49] Freire Santana M Borba MGS Baía-da-Silva DC Val F Alexandre MAA Brito-Sousa JD. Case report: adrenal pathology findings in severe COVID-19: an autopsy study. Am J Trop Med hygiene. (2020) 103:1604–7. doi: 10.4269/ajtmh.20-0787 PMC7543860 32876012

[50] Zinserling VA Semenova NY Markov AG Rybalchenko OV Wang J Rodionov RN. Inflammatory cell infiltration of adrenals in COVID-19. Hormone Metab Res = Hormon- und Stoffwechselforschung = Hormones metabolisme. (2020) 52:639–41. doi: 10.1055/a-1191-8094 32629518

[51] Alzahrani AS Mukhtar N Aljomaiah A Aljamei H Bakhsh A AlSudani N. The impact of COVID-19 viral infection on the hypothalamic-pituitary-adrenal axis. Endocrine practice: Off J Am Coll Endocrinol Am Assoc Clin Endocrinologists. (2021) 27:83–9. doi: 10.1016/j.eprac.2020.10.014 PMC7837186 33554871

[52] Ekinci I Hursitoglu M Tunc M Kazezoglu C Isiksacan N Yurt S. Adrenocortical system hormones in non-critically ill COVID-19 patients. Acta endocrinologica. (2021) 17:83–9. doi: 10.4183/aeb.2021.83 PMC8417496 34539914

[53] Kaya MG Alanli R Kucukay MB Ulukaya FB Bakir F. PITUITARY FUNCTIONS AFTER RECOVERY FROM COVID-19. Acta endocrinologica (Bucharest Romania: 2005). (2023) 19:314–8. doi: 10.4183/aeb.2023.314 PMC10863953 38356979

[54] Gonen MS De Bellis A Durcan E Bellastella G Cirillo P Scappaticcio L. Assessment of neuroendocrine changes and hypothalamo-pituitary autoimmunity in patients with COVID-19. Hormone Metab Res = Hormon- und Stoffwechselforschung = Hormones metabolisme. (2022) 54:153–61. doi: 10.1055/a-1764-1260 35276740

[55] Wheatland R. Molecular mimicry of ACTH in SARS - implications for corticosteroid treatment and prophylaxis. Med hypotheses. (2004) 63:855–62. doi: 10.1016/j.mehy.2004.04.009 PMC7126000 15488660

[56] Téblick A Langouche L Van den Berghe G. Anterior pituitary function in critical illness. Endocrine connections. (2019) 8:R131–r43. doi: 10.1530/EC-19-0318 PMC6709544 31340197

[57] Nicolaides NC Charmandari E Kino T Chrousos GP. Stress-related and circadian secretion and target tissue actions of glucocorticoids: impact on health. Front endocrinology. (2017) 8:70. doi: 10.3389/fendo.2017.00070 PMC5408025 28503165

[58] McGowan M Lim S O’Reilly SL Harrison CL Enticott J Teede H. Impact of COVID-19 restriction on weight, physical activity, diet and psychological distress on people with PCOS. Nutrients. (2023) 15(11):2579. doi: 10.3390/nu15112579 37299542 PMC10255147

[59] Bakaloudi DR Barazzoni R Bischoff SC Breda J Wickramasinghe K Chourdakis M. Impact of the first COVID-19 lockdown on body weight: A combined systematic review and a meta-analysis. Clin Nutr (Edinburgh Scotland). (2022) 41:3046–54. doi: 10.1016/j.clnu.2021.04.015 PMC8056819 34049749

[60] Jenssen BP Kelly MK Powell M Bouchelle Z Mayne SL Fiks AG. COVID-19 and changes in child obesity. Pediatrics. (2021) 147(5):e2021050123. doi: 10.1542/peds.2021-050123 33653879

[61] Tseng YH. Adipose tissue in communication: within and without. Nat Rev Endocrinology. (2023) 19:70–1. doi: 10.1038/s41574-022-00789-x PMC9756712 36526875

[62] Saccon TD Mousovich-Neto F Ludwig RG Carregari VC Dos Anjos Souza AB Dos Passos ASC. SARS-CoV-2 infects adipose tissue in a fat depot- and viral lineage-dependent manner. Nat Commun. (2022) 13:5722. doi: 10.1038/s41467-022-33218-8 36175400 PMC9521555

[63] Kruglikov IL Scherer PE. The role of adipocytes and adipocyte-like cells in the severity of COVID-19 infections. Obes (Silver Spring Md). (2020) 28:1187–90. doi: 10.1002/oby.22856 PMC7267593 32339391

[64] Al Heialy S Hachim MY Senok A Gaudet M Abou Tayoun A Hamoudi R. Regulation of angiotensin- converting enzyme 2 in obesity: implications for COVID-19. Front Physiol. (2020) 11:555039. doi: 10.3389/fphys.2020.555039 33071815 PMC7531362

[65] Quaranta P Scabia G Storti B Dattilo A Quintino L Perrera P. SARS-coV-2 infection alters the phenotype and gene expression of adipocytes. Int J Mol Sci. (2024) 25(4):2086. doi: 10.3390/ijms25042086 38396763 PMC10889321

[66] Glueck CJ Goldenberg N. Characteristics of obesity in polycystic ovary syndrome: Etiology, treatment, and genetics. Metabolism: Clin experimental. (2019) 92:108–20. doi: 10.1016/j.metabol.2018.11.002 30445140

[67] Pasquali R Pelusi C Genghini S Cacciari M Gambineri A. Obesity and reproductive disorders in women. Hum Reprod update. (2003) 9:359–72. doi: 10.1093/humupd/dmg024 12926529

[68] Silvestris E de Pergola G Rosania R Loverro G. Obesity as disruptor of the female fertility. Reprod Biol endocrinology: RB&E. (2018) 16:22. doi: 10.1186/s12958-018-0336-z 29523133 PMC5845358

[69] Littlejohn EE Weiss RE Deplewski D Edidin DV Rosenfield R. Intractable early childhood obesity as the initial sign of insulin resistant hyperinsulinism and precursor of polycystic ovary syndrome. J Pediatr Endocrinol metabolism: JPEM. (2007) 20:41–51. doi: 10.1515/JPEM.2007.20.1.41 17315528

[70] Christensen SB Black MH Smith N Martinez MM Jacobsen SJ Porter AH. Prevalence of polycystic ovary syndrome in adolescents. Fertility sterility. (2013) 100:470–7. doi: 10.1016/j.fertnstert.2013.04.001 PMC3813299 23756098

[71] Goodarzi MO Erickson S Port SC Jennrich RI Korenman SG. beta-Cell function: a key pathological determinant in polycystic ovary syndrome. J Clin Endocrinol Metab. (2005) 90:310–5. doi: 10.1210/jc.2004-1006 15507511

[72] Groop LC Bonadonna RC DelPrato S Ratheiser K Zyck K Ferrannini E. Glucose and free fatty acid metabolism in non-insulin-dependent diabetes mellitus. Evidence for multiple sites of insulin resistance. J Clin Invest. (1989) 84:205–13. doi: 10.1172/JCI114142 PMC303971 2661589

[73] Wu L Girgis CM Cheung NW. COVID-19 and diabetes: Insulin requirements parallel illness severity in critically unwell patients. Clin endocrinology. (2020) 93:390–3. doi: 10.1111/cen.14288 PMC7404426 32683745

[74] Šestan M Marinović S Kavazović I Cekinović Đ Wueest S Turk Wensveen T. Virus-induced interferon-γ Causes insulin resistance in skeletal muscle and derails glycemic control in obesity. Immunity. (2018) 49:164–77.e6. doi: 10.1016/j.immuni.2018.05.005 29958802

[75] de Carvalho Vidigal F Guedes Cocate P Gonçalves Pereira L de Cássia Gonçalves Alfenas R. The role of hyperglycemia in the induction of oxidative stress and inflammatory process. Nutricion hospitalaria. (2012) 27:1391–8. doi: 10.3305/nh.2012.27.5.5917 23478683

[76] Solerte SB D’Addio F Trevisan R Lovati E Rossi A Pastore I. Sitagliptin treatment at the time of hospitalization was associated with reduced mortality in patients with type 2 diabetes and COVID-19: A multicenter, case-control, retrospective, observational study. Diabetes Care. (2020) 43:2999–3006. doi: 10.2337/dc20-1521 32994187 PMC7770266

[77] Sardu C D’Onofrio N Balestrieri ML Barbieri M Rizzo MR Messina V. Outcomes in patients with hyperglycemia affected by COVID-19: can we do more on glycemic control? Diabetes Care. (2020) 43:1408–15. doi: 10.2337/dc20-0723 PMC7305003 32430456

[78] Montefusco L Ben Nasr M D’Addio F Loretelli C Rossi A Pastore I. Acute and long-term disruption of glycometabolic control after SARS-CoV-2 infection. Nat Metab. (2021) 3:774–85. doi: 10.1038/s42255-021-00407-6 PMC9931026 34035524

[79] Ramakrishnan RK Kashour T Hamid Q Halwani R Tleyjeh IM. Unraveling the mystery surrounding post-acute sequelae of COVID-19. Front Immunol. (2021) 12:686029. doi: 10.3389/fimmu.2021.686029 34276671 PMC8278217

[80] Chen M Zhu B Chen D Hu X Xu X Shen WJ. COVID-19 may increase the risk of insulin resistance in adult patients without diabetes: A 6-month prospective study. Endocrine practice: Off J Am Coll Endocrinol Am Assoc Clin Endocrinologists. (2021) 27:834–41. doi: 10.1016/j.eprac.2021.04.004 PMC8054613 33887468

[81] Tremellen K Pearce K. Dysbiosis of Gut Microbiota (DOGMA)–a novel theory for the development of Polycystic Ovarian Syndrome. Med hypotheses. (2012) 79:104–12. doi: 10.1016/j.mehy.2012.04.016 22543078

[82] Zhou L Ni Z Cheng W Yu J Sun S Zhai D. Characteristic gut microbiota and predicted metabolic functions in women with PCOS. Endocrine connections. (2020) 9:63–73. doi: 10.1530/EC-19-0522 31972546 PMC6993273

[83] Yeoh YK Zuo T Lui GC Zhang F Liu Q Li AY. Gut microbiota composition reflects disease severity and dysfunctional immune responses in patients with COVID-19. Gut. (2021) 70:698–706. doi: 10.1136/gutjnl-2020-323020 33431578 PMC7804842

[84] Camargo SM Singer D Makrides V Huggel K Pos KM Wagner CA. Tissue-specific amino acid transporter partners ACE2 and collectrin differentially interact with hartnup mutations. Gastroenterology. (2009) 136:872–82. doi: 10.1053/j.gastro.2008.10.055 PMC7094282 19185582

[85] Perlot T Penninger JM. ACE2 - from the renin-angiotensin system to gut microbiota and malnutrition. Microbes infection. (2013) 15:866–73. doi: 10.1016/j.micinf.2013.08.003 PMC7110844 23962453

[86] Yonker LM Gilboa T Ogata AF Senussi Y Lazarovits R Boribong BP. Multisystem inflammatory syndrome in children is driven by zonulin-dependent loss of gut mucosal barrier. J Clin Invest. (2021) 131(14):e149633. doi: 10.1172/JCI149633 34032635 PMC8279585

[87] Han MS White A Perry RJ Camporez JP Hidalgo J Shulman GI. Regulation of adipose tissue inflammation by interleukin 6. Proc Natl Acad Sci United States America. (2020) 117:2751–60. doi: 10.1073/pnas.1920004117 PMC7022151 31980524

[88] Sbierski-Kind J Mai K Kath J Jurisch A Streitz M Kuchenbecker L. Association between subcutaneous adipose tissue inflammation, insulin resistance, and calorie restriction in obese females. J Immunol (Baltimore Md: 1950). (2020) 205:45–55. doi: 10.4049/jimmunol.2000108 32482712

[89] Zheng M Gao Y Wang G Song G Liu S Sun D. Functional exhaustion of antiviral lymphocytes in COVID-19 patients. Cell Mol Immunol. (2020) 17:533–5. doi: 10.1038/s41423-020-0402-2 PMC7091858 32203188

[90] Zheng HY Zhang M Yang CX Zhang N Wang XC Yang XP. Elevated exhaustion levels and reduced functional diversity of T cells in peripheral blood may predict severe progression in COVID-19 patients. Cell Mol Immunol. (2020) 17:541–3. doi: 10.1038/s41423-020-0401-3 PMC7091621 32203186

[91] De Biasi S Meschiari M Gibellini L Bellinazzi C Borella R Fidanza L. Marked T cell activation, senescence, exhaustion and skewing towards TH17 in patients with COVID-19 pneumonia. Nat Commun. (2020) 11:3434. doi: 10.1038/s41467-020-17292-4 32632085 PMC7338513

[92] Sun X Wang T Cai D Hu Z Chen J Liao H. Cytokine storm intervention in the early stages of COVID-19 pneumonia. Cytokine Growth factor Rev. (2020) 53:38–42. doi: 10.1016/j.cytogfr.2020.04.002 32360420 PMC7182527

[93] Afrin LB Weinstock LB Molderings GJ. Covid-19 hyperinflammation and post-covid-19 illness may be rooted in mast cell activation syndrome. Int J Infect diseases: IJID: Off Publ Int Soc Infect Diseases. (2020) 100:327–32. doi: 10.1016/j.ijid.2020.09.016 PMC7529115 32920235

[94] Merad M Blish CA Sallusto F Iwasaki A. The immunology and immunopathology of COVID-19. Sci (New York NY). (2022) 375:1122–7. doi: 10.1126/science.abm8108 PMC12828912 35271343

[95] Besnier F Bérubé B Malo J Gagnon C Grégoire CA Juneau M. Cardiopulmonary rehabilitation in long-COVID-19 patients with persistent breathlessness and fatigue: the COVID-rehab study. Int J Environ Res Public Health. (2022) 19(7):4133. doi: 10.3390/ijerph19074133 35409815 PMC8998214

[96] Carfì A Bernabei R Landi F. Persistent symptoms in patients after acute COVID-19. Jama. (2020) 324:603–5. doi: 10.1001/jama.2020.12603 PMC7349096 32644129

[97] Bellan M Soddu D Balbo PE Baricich A Zeppegno P Avanzi GC. Respiratory and psychophysical sequelae among patients with COVID-19 four months after hospital discharge. JAMA network Open. (2021) 4:e2036142. doi: 10.1001/jamanetworkopen.2020.36142 33502487 PMC7841464

[98] Lenarcik A Bidzińska-Speichert B. Cardiopulmonary functional capacity and the role of exercise in improving maximal oxygen consumption in women with PCOS. Endokrynologia Polska. (2010) 61:207–9. doi: 10.1016/j.ecl.2009.10.010 20464708

[99] Lazarus R Sparrow D Weiss ST. Baseline ventilatory function predicts the development of higher levels of fasting insulin and fasting insulin resistance index: the Normative Aging Study. Eur Respir J. (1998) 12:641–5. doi: 10.1183/09031936.98.12030641 9762793

[100] Ford ES Mannino DM. Prospective association between lung function and the incidence of diabetes: findings from the National Health and Nutrition Examination Survey Epidemiologic Follow-up Study. Diabetes Care. (2004) 27:2966–70. doi: 10.2337/diacare.27.12.2966 15562215

[101] Zaigham S Nilsson PM Wollmer P Engström G. The temporal relationship between poor lung function and the risk of diabetes. BMC pulmonary Med. (2016) 16:75. doi: 10.1186/s12890-016-0227-z 27165091 PMC4863358

[102] Engström G Hedblad B Nilsson P Wollmer P Berglund G Janzon L. Lung function, insulin resistance and incidence of cardiovascular disease: a longitudinal cohort study. J Internal Med. (2003) 253:574–81. doi: 10.1046/j.1365-2796.2003.01138.x 12702035

[103] Jankowich M Elston B Liu Q Abbasi S Wu WC Blackshear C. Restrictive spirometry pattern, cardiac structure and function, and incident heart failure in african americans. Jackson Heart Study Ann Am Thorac Society. (2018) 15:1186–96. doi: 10.1513/AnnalsATS.201803-184OC PMC6321994 30011374

[104] Braun B Rock PB Zamudio S Wolfel GE Mazzeo RS Muza SR. Women at altitude: short-term exposure to hypoxia and/or alpha(1)-adrenergic blockade reduces insulin sensitivity. J Appl Physiol (Bethesda Md: 1985). (2001) 91:623–31. doi: 10.1152/jappl.2001.91.2.623 11457773

[105] Schmidt MI Duncan BB Sharrett AR Lindberg G Savage PJ Offenbacher S. Markers of inflammation and prediction of diabetes mellitus in adults (Atherosclerosis Risk in Communities study): a cohort study. Lancet (London England). (1999) 353:1649–52. doi: 10.1016/s0140-6736(99)01046-6 10335783

[106] Semenza GL. Oxygen-regulated transcription factors and their role in pulmonary disease. Respir Res. (2000) 1:159–62. doi: 10.1186/rr27 PMC59554 11667980

[107] Orvieto R Segev-Zahav A Aizer A. Does COVID-19 infection influence patients’ performance during IVF-ET cycle?: an observational study. Gynecological endocrinology: Off J Int Soc Gynecological Endocrinology. (2021) 37:895–7. doi: 10.1080/09513590.2021.1918080 33974475

[108] Youngster M Avraham S Yaakov O Landau Rabbi M Gat I Yerushalmi G. IVF under COVID-19: treatment outcomes of fresh ART cycles. Hum Reprod (Oxford England). (2022) 37:947–53. doi: 10.1093/humrep/deac043 PMC8903458 35212741

[109] Wang M Hu J Huang B Yang Q Liu S Li Z. Investigating the impact of SARS-CoV-2 infection on basic semen parameters and *in vitro* fertilization/intracytoplasmic sperm injection outcomes: a retrospective cohort study. Reprod Biol endocrinology: RB&E. (2022) 20:46. doi: 10.1186/s12958-022-00918-1 35260151 PMC8901866

[110] Veiga A Gianaroli L Ory S Horton M Feinberg E Penzias A. Assisted reproduction and COVID-19: a joint statement of ASRM, ESHRE, and IFFS. Global Reprod Health. (2020) 5:10.1097. doi: 10.1093/hropen/hoaa033 PMC7513957 34192221

[111] Boivin J Harrison C Mathur R Burns G Pericleous-Smith A Gameiro S. Patient experiences of fertility clinic closure during the COVID-19 pandemic: appraisals, coping and emotions. Hum Reprod (Oxford England). (2020) 35:2556–66. doi: 10.1093/humrep/deaa218 PMC7454659 32761248

[112] Esposito V Rania E Lico D Pedri S Fiorenza A Strati MF. Influence of COVID-19 pandemic on the psychological status of infertile couples. Eur J obstetrics gynecology Reprod Biol. (2020) 253:148–53. doi: 10.1016/j.ejogrb.2020.08.025 PMC7443353 32866858

[113] Bhattacharya S Maheshwari A Ratna MB van Eekelen R Mol BW McLernon DJ. Prioritizing IVF treatment in the post-covid 19 era: a predictive modelling study based on UK national data. Hum Reprod (Oxford England). (2021) 36:666–75. doi: 10.1093/humrep/deaa339 PMC7717242 33226080

[114] Chandi A Jain N. State of assisted reproduction technology in the coronavirus disease 2019 era and consequences on human reproductive system. Biol reproduction. (2021) 105:808–21. doi: 10.1093/biolre/ioab122 34159367

[115] Lam JS Shere M Motamedi N Vilos GA Abu-Rafea B Vilos AG. Impact of the COVID-19 pandemic on access to fertility care: A retrospective study at a university-affiliated fertility practice. J obstetrics gynaecology Canada: JOGC = J d’obstetrique gynecologie du Canada: JOGC. (2022) 44:378–82. doi: 10.1016/j.jogc.2021.10.017 PMC8610176 34749024

[116] Gromski PS Smith A Lawlor DA Sharara FI Nelson SM. 2008 financial crisis versus 2020 economic fallout: how COVID-19 might influence fertility treatment and live births. Reprod biomedicine Online. (2021) 42:1087–96. doi: 10.1016/j.rbmo.2021.03.017 33931369

[117] Kyrou I Karteris E Robbins T Chatha K Drenos F Randeva HS. Polycystic ovary syndrome (PCOS) and COVID-19: an overlooked female patient population at potentially higher risk during the COVID-19 pandemic. BMC Med. (2020) 18:220. doi: 10.1186/s12916-020-01697-5 32664957 PMC7360476

[118] Li W Yu N Kang Q Zeng W Deng D Chen S. Clinical manifestations and maternal and perinatal outcomes with COVID-19. Am J Reprod Immunol (New York NY: 1989). (2020) 84:e13340. doi: 10.1111/aji.13340 32894803

[119] DeSisto CL Wallace B Simeone RM Polen K Ko JY Meaney-Delman D. Risk for stillbirth among women with and without COVID-19 at delivery hospitalization - United States, march 2020-september 2021. MMWR Morbidity mortality weekly Rep. (2021) 70:1640–5. doi: 10.15585/mmwr.mm7047e1 PMC8612508 34818318

[120] Zaigham M Andersson O. Maternal and perinatal outcomes with COVID-19: A systematic review of 108 pregnancies. Acta obstetricia gynecologica Scandinavica. (2020) 99:823–9. doi: 10.1111/aogs.13867 PMC7262097 32259279

[121] Allotey J Stallings E Bonet M Yap M Chatterjee S Kew T. Clinical manifestations, risk factors, and maternal and perinatal outcomes of coronavirus disease 2019 in pregnancy: living systematic review and meta-analysis. BMJ. (2020) 370:m3320. doi: 10.1136/bmj.m3320 32873575 PMC7459193

[122] Kotlyar AM Grechukhina O Chen A Popkhadze S Grimshaw A Tal O. Vertical transmission of coronavirus disease 2019: a systematic review and meta-analysis. Am J obstetrics gynecology. (2021) 224:35–53.e3. doi: 10.1016/j.ajog.2020.07.049 PMC7392880 32739398

[123] Penfield CA Brubaker SG Limaye MA Lighter J Ratner AJ Thomas KM. Detection of severe acute respiratory syndrome coronavirus 2 in placental and fetal membrane samples. Am J obstetrics gynecology MFM. (2020) 2:100133. doi: 10.1016/j.ajogmf.2020.100133 PMC7205635 32391518

[124] Ashary N Bhide A Chakraborty P Colaco S Mishra A Chhabria K. Single-cell RNA-seq identifies cell subsets in human placenta that highly expresses factors driving pathogenesis of SARS-coV-2. Front Cell Dev Biol. (2020) 8:783. doi: 10.3389/fcell.2020.00783 32974340 PMC7466449

[125] Egloff C Vauloup-Fellous C Picone O Mandelbrot L Roques P. Evidence and possible mechanisms of rare maternal-fetal transmission of SARS-CoV-2. J Clin virology: Off Publ Pan Am Soc Clin Virology. (2020) 128:104447. doi: 10.1016/j.jcv.2020.104447 PMC7233246 32425663

[126] Jafari M Pormohammad A Sheikh Neshin SA Ghorbani S Bose D Alimohammadi S. Clinical characteristics and outcomes of pregnant women with COVID-19 and comparison with control patients: A systematic review and meta-analysis. Rev Med virology. (2021) 31:1–16. doi: 10.1002/rmv.2208 PMC7883245 33387448

[127] Kumar J Meena J Yadav A Kumar P. SARS-CoV-2 detection in human milk: a systematic review. J maternal-fetal neonatal medicine: Off J Eur Assoc Perinatal Medicine Fed Asia Oceania Perinatal Societies Int Soc Perinatal Obstet. (2022) 35:5456–63. doi: 10.1080/14767058.2021.1882984 33550866

[128] Norman M Navér L Söderling J Ahlberg M Hervius Askling H Aronsson B. Association of maternal SARS-coV-2 infection in pregnancy with neonatal outcomes. Jama. (2021) 325:2076–86. doi: 10.1001/jama.2021.5775 PMC8085767 33914014

[129] Teede HJ Tay CT Laven JJE Dokras A Moran LJ Piltonen TT. Recommendations from the 2023 international evidence-based guideline for the assessment and management of polycystic ovary syndrome. J Clin Endocrinol Metab. (2023) 108:2447–69. doi: 10.1210/clinem/dgad463 PMC10505534 37580314

[130] Wilder-Smith A Freedman DO. Isolation, quarantine, social distancing and community containment: pivotal role for old-style public health measures in the novel coronavirus (2019-nCoV) outbreak. J travel Med. (2020) 27(2):taaa020. doi: 10.1093/jtm/taaa020 32052841 PMC7107565

[131] Khan MA Moverley Smith JE. Covibesity,” a new pandemic. Obes Med. (2020) 19:100282. doi: 10.1016/j.obmed.2020.100282 32835125 PMC7371584

[132] Arnold DT Hamilton FW Milne A Morley AJ Viner J Attwood M. Patient outcomes after hospitalisation with COVID-19 and implications for follow-up: results from a prospective UK cohort. Thorax. (2021) 76:399–401. doi: 10.1136/thoraxjnl-2020-216086 33273026 PMC7716340

[133] Sykes DL Holdsworth L Jawad N Gunasekera P Morice AH Crooks MG. Post-covid-19 symptom burden: what is long-COVID and how should we manage it? Lung. (2021) 199:113–9. doi: 10.1183/13993003.congress-2021.OA4189 PMC7875681 33569660

[134] Eyupoglu ND Aksun S Ozturk M Yildiz BO. Impact of social isolation during COVID-19 pandemic on health behaviors and weight management in women with polycystic ovary syndrome. Eating weight disorders: EWD. (2022) 27:2407–13. doi: 10.1007/s40519-022-01369-8 PMC8865179 35195885

[135] Yang Y Deng H Li T Xia M Liu C Bu XQ. The mental health of Chinese women with polycystic ovary syndrome is related to sleep disorders, not disease status. J Affect Disord. (2021) 282:51–7. doi: 10.1016/j.jad.2020.12.084 33388474

[136] Minkel JD Banks S Htaik O Moreta MC Jones CW McGlinchey EL. Sleep deprivation and stressors: evidence for elevated negative affect in response to mild stressors when sleep deprived. Emotion. (2012) 12:1015–20. doi: 10.1037/a0026871 PMC3964364 22309720

[137] Vgontzas AN Zoumakis E Bixler EO Lin HM Follett H Kales A. Adverse effects of modest sleep restriction on sleepiness, performance, and inflammatory cytokines. J Clin Endocrinol Metab. (2004) 89:2119–26. doi: 10.1210/jc.2003-031562 15126529

[138] Leproult R Van Cauter E. Role of sleep and sleep loss in hormonal release and metabolism. Endocrine Dev. (2010) 17:11–21. doi: 10.1159/000262524 PMC3065172 19955752

[139] Knutson KL Van Cauter E. Associations between sleep loss and increased risk of obesity and diabetes. Ann New York Acad Sci. (2008) 1129:287–304. doi: 10.1196/annals.1417.033 18591489 PMC4394987

[140] Van Cauter E Holmback U Knutson K Leproult R Miller A Nedeltcheva A. Impact of sleep and sleep loss on neuroendocrine and metabolic function. Hormone Res. (2007) 67 Suppl 1:2–9. doi: 10.1159/000097543 17308390

[141] Mizgier M Jarząbek-Bielecka G Opydo-Szymaczek J Wendland N Więckowska B Kędzia W. Risk factors of overweight and obesity related to diet and disordered eating attitudes in adolescent girls with clinical features of polycystic ovary syndrome. J Clin Med. (2020) 9(9):3041. doi: 10.3390/jcm9093041 32967289 PMC7564079

[142] Barrea L Arnone A Annunziata G Muscogiuri G Laudisio D Salzano C. Adherence to the mediterranean diet, dietary patterns and body composition in women with polycystic ovary syndrome (PCOS). Nutrients. (2019) 11(10):2278. doi: 10.3390/nu11102278 31547562 PMC6836220

[143] Parker J O’Brien C Gersh FL. Developmental origins and transgenerational inheritance of polycystic ovary syndrome. Aust New Z J obstetrics gynaecology. (2021) 61:922–6. doi: 10.1111/ajo.13420 34403138

[144] Maestre A Sospedra I Martínez-Sanz JM Gutierrez-Hervas A Fernández-Saez J Hurtado-Sánchez JA. Assessment of spanish food consumption patterns during COVID-19 home confinement. Nutrients. (2021) 13(11):4122. doi: 10.3390/nu13114122 34836377 PMC8617653

[145] Gibson EL. Emotional influences on food choice: sensory, physiological and psychological pathways. Physiol behavior. (2006) 89:53–61. doi: 10.1016/j.physbeh.2006.01.024 16545403

[146] Rodríguez-Martín BC Meule A. Food craving: new contributions on its assessment, moderators, and consequences. Front Psychol. (2015) 6:21. doi: 10.3389/fpsyg.2015.00021 25657636 PMC4302707

[147] Rauber F da Costa Louzada ML Steele EM Millett C Monteiro CA Levy RB. Ultra-processed food consumption and chronic non-communicable diseases-related dietary nutrient profile in the UK (2008–2014). Nutrients. (2018) 10(5):587. doi: 10.3390/nu10050587 29747447 PMC5986467

[148] Ma Y Ratnasabapathy R Gardiner J. Carbohydrate craving: not everything is sweet. Curr Opin Clin Nutr Metab Care. (2017) 20:261–5. doi: 10.1097/MCO.0000000000000374 PMC5837018 28375878

[149] Silva Meneguelli T Viana Hinkelmann J Hermsdorff HHM Zulet M Martínez JA Bressan J. Food consumption by degree of processing and cardiometabolic risk: a systematic review. Int J Food Sci Nutr. (2020) 71:678–92. doi: 10.1080/09637486.2020.1725961 32053758

[150] Muscogiuri G Pugliese G Barrea L Savastano S Colao A. Commentary: obesity: the “Achilles heel” for COVID-19? Metabolism: Clin Exp. (2020) 108:154251. doi: 10.1016/j.metabol.2020.154251 PMC7184987 32353356

[151] Lee I Cooney LG Saini S Smith ME Sammel MD Allison KC. Increased risk of disordered eating in polycystic ovary syndrome. Fertility sterility. (2017) 107:796–802. doi: 10.1016/j.fertnstert.2016.12.014 28104244

[152] Ma R Zou Y Wang W Zheng Q Feng Y Dong H. Obesity management in polycystic ovary syndrome: disparity in knowledge between obstetrician-gynecologists and reproductive endocrinologists in China. BMC endocrine Disord. (2021) 21:182. doi: 10.1186/s12902-021-00848-w PMC8422662 34488736

[153] Teede HJ Misso ML Costello MF Dokras A Laven J Moran L. Recommendations from the international evidence-based guideline for the assessment and management of polycystic ovary syndrome. Hum Reprod (Oxford England). (2018) 33:1602–18. doi: 10.1093/humrep/dey256 PMC6112576 30052961

[154] Gibson-Helm M Dokras A Karro H Piltonen T Teede HJ. Knowledge and practices regarding polycystic ovary syndrome among physicians in europe, north america, and internationally: an online questionnaire-based study. Semin Reprod Med. (2018) 36:19–27. doi: 10.1055/s-0038-1667155 30189447

[155] Dokras A Saini S Gibson-Helm M Schulkin J Cooney L Teede H. Gaps in knowledge among physicians regarding diagnostic criteria and management of polycystic ovary syndrome. Fertility sterility. (2017) 107:1380–6.e1. doi: 10.1016/j.fertnstert.2017.04.011 28483503

[156] Tomlinson J Pinkney J Adams L Stenhouse E Bendall A Corrigan O. The diagnosis and lived experience of polycystic ovary syndrome: A qualitative study. J advanced nursing. (2017) 73:2318–26. doi: 10.1111/jan.13300 28329428

[157] Jeong Y Crowell T Devon-Sand A Sakata T Sattler A Shah S. Building pandemic-resilient primary care systems: lessons learned from COVID-19. J Med Internet Res. (2024) 26:e47667. doi: 10.2196/47667 38393776 PMC10924259

[158] Williams S Tsiligianni I. COVID-19 poses novel challenges for global primary care. NPJ primary Care Respir Med. (2020) 30:30. doi: 10.1038/s41533-020-0187-x 32555160 PMC7303134

[159] Lee IT Sansone S Irfan M Copp T Beidas R Dokras A. Implementation of international guidelines for polycystic ovary syndrome: barriers and facilitators among gynecologists and primary care providers. F&S Rep. (2022) 3:94–101. doi: 10.1016/j.xfre.2022.01.005 PMC9250120 35789712

[160] Hillman SC Bryce C Caleyachetty R Dale J. Women’s experiences of diagnosis and management of polycystic ovary syndrome: a mixed-methods study in general practice. Br J Gen practice: J R Coll Gen Practitioners. (2020) 70:e322–e9. doi: 10.3399/bjgp20X708881 PMC7065681 32152043

[161] Moreno C Wykes T Galderisi S Nordentoft M Crossley N Jones N. How mental health care should change as a consequence of the COVID-19 pandemic. Lancet Psychiatry. (2020) 7:813–24. doi: 10.1016/S2215-0366(20)30307-2 PMC7365642 32682460

[162] Sydora BC Wilke MS McPherson M Chambers S Ghosh M Vine DF. Challenges in diagnosis and health care in polycystic ovary syndrome in Canada: a patient view to improve health care. BMC women’s Health. (2023) 23:569. doi: 10.1186/s12905-023-02732-2 37925392 PMC10625259

[163] Kite C Atkinson L McGregor G Clark CCT Brown JE Kyrou I. Sleep disruption and depression, stress and anxiety levels in women with polycystic ovary syndrome (PCOS) during the lockdown measures for COVID-19 in the UK. Front Global women’s Health. (2021) 2:649104. doi: 10.3389/fgwh.2021.649104 PMC8593975 34816205

[164] Atkinson L Kite C McGregor G James T Clark CCT Randeva HS. Uncertainty, anxiety and isolation: experiencing the COVID-19 pandemic and lockdown as a woman with polycystic ovary syndrome (PCOS). J personalized Med. (2021) 11(10):952. doi: 10.3390/jpm11100952 PMC8539750 34683093

[165] Giannopoulou I Tsobanoglou GO. COVID-19 pandemic: challenges and opportunities for the Greek health care system. Irish J psychol Med. (2020) 37:226–30. doi: 10.1017/ipm.2020.35 PMC7287301 32406360

[166] Emanuel EJ Persad G Upshur R Thome B Parker M Glickman A. Fair allocation of scarce medical resources in the time of covid-19. New Engl J Med. (2020) 382:2049–55. doi: 10.1056/NEJMsb2005114 32202722

[167] Craighead CG Collart C Frankel R Rose S Misra-Hebert AD Tucker Edmonds B. Impact of telehealth on the delivery of prenatal care during the COVID-19 pandemic: mixed methods study of the barriers and opportunities to improve health care communication in discussions about pregnancy and prenatal genetic testing. JMIR formative Res. (2022) 6:e38821. doi: 10.2196/38821 PMC9728023 36383634

[168] Dulaimy K Pham RH Farag A. The impact of COVID on health systems: the workforce and telemedicine perspective. Semin ultrasound CT MR. (2024) 45:314–317. doi: 10.1053/j.sult.2024.03.002 38527671

[169] Dwomoh D Yeboah I Ndejjo R Kabwama SN Aheto JM Liu A. COVID-19 outbreak control strategies and their impact on the provision of essential health services in Ghana: An exploratory-sequential study. PloS One. (2023) 18:e0279528. doi: 10.1371/journal.pone.0279528 37972045 PMC10653447

[170] Amu H Dowou RK Boateng LA Tarkang EE. Implications of COVID-19 for the management of chronic non-communicable diseases in sub-Saharan Africa: application of the chronic care model. Pan Afr Med J. (2020) 35:94. doi: 10.11604/pamj.supp.2020.35.24047 PMC7875795 33623618

[171] Aborode AT Fajemisin EA Aiyenuro EA Alakitan MT Ariwoola MO Imisioluwa JO. Neglected tropical diseases (NTDs) and COVID-19 pandemic in africa: special focus on control strategies. Combinatorial Chem High throughput screening. (2022) 25:2387–90. doi: 10.2174/1386207325666220427123349 35490317

[172] Beyene DT Lemma KT Sultan SA Sinbiro IA. Perceived impact of COVID-19 on routine care of patients with chronic non-communicable diseases: a cross-sectional study. Pan Afr Med J. (2022) 43:212. doi: 10.11604/pamj.2022.43.212.32769 36974310 PMC10038762

[173] Owopetu O Fasehun LK Abakporo U. COVID-19: implications for NCDs and the continuity of care in Sub-Saharan Africa. Global Health promotion. (2021) 28:83–6. doi: 10.1177/1757975921992693 33579179

[174] Wright A Salazar A Mirica M Volk LA Schiff GD. The invisible epidemic: neglected chronic disease management during COVID-19. J Gen Internal Med. (2020) 35:2816–7. doi: 10.1007/s11606-020-06025-4 PMC7359916 32666485

[175] Damone AL Joham AE Loxton D Earnest A Teede HJ Moran LJ. Depression, anxiety and perceived stress in women with and without PCOS: a community-based study. psychol Med. (2019) 49:1510–20. doi: 10.1017/S0033291718002076 30131078

[176] Tippett A. Life on pause: An analysis of UK fertility patients’ coping mechanisms after the cancellation of fertility treatment due to COVID-19. J Health Psychol. (2022) 27:1583–600. doi: 10.1177/1359105321999711 PMC9092918 33685265

[177] Ramezani M Takian A Bakhtiari A Rabiee HR Ghazanfari S Sazgarnejad S. Research agenda for using artificial intelligence in health governance: interpretive scoping review and framework. BioData mining. (2023) 16:31. doi: 10.1186/s13040-023-00346-w 37904172 PMC10617108

[178] Jonnagaddala J Godinho MA Liaw ST. From telehealth to virtual primary care in Australia? A Rapid scoping review. Int J Med informatics. (2021) 151:104470. doi: 10.1016/j.ijmedinf.2021.104470 PMC9761082 34000481

[179] Byrne A Barber R Lim CH. Impact of the COVID-19 pandemic – a mental health service perspective. Prog Neurol Psychiatry. (2021) 25:27–33b. doi: 10.1002/pnp.708

[180] Walker A Alkasaby MA Baingana F Bosu WK Abdulaziz M Westerveld R. Challenges and opportunities for mental health and psychosocial support in the COVID-19 response in africa: A mixed-methods study. Int J Environ Res Public Health. (2022) 19(15):9313. doi: 10.3390/ijerph19159313 35954669 PMC9368294

[181] Shoib S Arif N Nahidi M Rumiyya K Swed S Yusha’u Armiya’u A. Nagorno-Karabakh conflict: Mental health repercussions and challenges in Azerbaijan. Asian J Psychiatry. (2022) 73:103095. doi: 10.1016/j.ajp.2022.103095 35468483

[182] Charlson F van Ommeren M Flaxman A Cornett J Whiteford H Saxena S. New WHO prevalence estimates of mental disorders in conflict settings: a systematic review and meta-analysis. Lancet (London England). (2019) 394:240–8. doi: 10.1016/S0140-6736(19)30934-1 PMC6657025 31200992

[183] Burki T. The indirect impact of COVID-19 on women. Lancet Infect diseases. (2020) 20:904–5. doi: 10.1016/S1473-3099(20)30568-5 PMC7836874 32738239

[184] Aksunger N Vernot C Littman R Voors M Meriggi NF Abajobir A. COVID-19 and mental health in 8 low- and middle-income countries: A prospective cohort study. PloS Med. (2023) 20:e1004081. doi: 10.1371/journal.pmed.1004081 37023021 PMC10079130

[185] Moitra M Owens S Hailemariam M Wilson KS Mensa-Kwao A Gonese G. Global mental health: where we are and where we are going. Curr Psychiatry Rep. (2023) 25:301–11. doi: 10.1007/s11920-023-01426-8 PMC10230139 37256471

[186] Zhang Y Lange KW. Coronavirus disease 2019 (COVID-19) and global mental health. Global Health J (Amsterdam Netherlands). (2021) 5:31–6. doi: 10.1016/j.glohj.2021.02.004 PMC7881705 33614179

[187] Semo BW Frissa SM. The mental health impact of the COVID-19 pandemic: implications for sub-saharan africa. Psychol Res Behav management. (2020) 13:713–20. doi: 10.2147/PRBM.S264286 PMC7508558 32982500

[188] Ruzzenenti G Maloberti A Giani V Biolcati M Leidi F Monticelli M. Covid and cardiovascular diseases: direct and indirect damages and future perspective. High Blood Pressure Cardiovasc prevention: Off J Ital Soc Hypertension. (2021) 28:439–45. doi: 10.1007/s40292-021-00464-8 PMC8233573 34173942

[189] Hemingway JF Singh N Starnes BW. Emerging practice patterns in vascular surgery during the COVID-19 pandemic. J Vasc surgery. (2020) 72:396–402. doi: 10.1016/j.jvs.2020.04.492 PMC7190553 32361072

[190] Blue R Yang AI Zhou C De Ravin E Teng CW Arguelles GR. Telemedicine in the era of coronavirus disease 2019 (COVID-19): A neurosurgical perspective. World neurosurgery. (2020) 139:549–57. doi: 10.1016/j.wneu.2020.05.066 PMC7229725 32426065

[191] Boyle JA Xu R Gilbert E Kuczynska-Burggraf M Tan B Teede H. Ask PCOS: identifying need to inform evidence-based app development for polycystic ovary syndrome. Semin Reprod Med. (2018) 36:59–65. doi: 10.1055/s-0038-1667187 30189452

[192] Wang L Liu Y Tan H Huang S. Transtheoretical model-based mobile health application for PCOS. Reprod Health. (2022) 19:117. doi: 10.1186/s12978-022-01422-w 35549736 PMC9097413

[193] Narang K Enninga EAL Gunaratne M Ibirogba ER Trad ATA Elrefaei A. SARS-coV-2 infection and COVID-19 during pregnancy: A multidisciplinary review. Mayo Clinic Proc. (2020) 95:1750–65. doi: 10.1016/j.mayocp.2020.05.011 PMC7260486 32753148

[194] Pountoukidou A Potamiti-Komi M Sarri V Papapanou M Routsi E Tsiatsiani AM. Management and prevention of COVID-19 in pregnancy and pandemic obstetric care: A review of current practices. Healthcare (Basel Switzerland). (2021) 9(4):467. doi: 10.3390/healthcare9040467 33920781 PMC8071177

[195] Ismayilova M Yaya S. What can be done to improve polycystic ovary syndrome (PCOS) healthcare? Insights from semi-structured interviews with women in Canada. BMC women’s Health. (2022) 22:157. doi: 10.1186/s12905-022-01734-w 35538531 PMC9092874

[196] Dokras A Stener-Victorin E Yildiz BO Li R Ottey S Shah D. Androgen Excess- Polycystic Ovary Syndrome Society: position statement on depression, anxiety, quality of life, and eating disorders in polycystic ovary syndrome. Fertility sterility. (2018) 109:888–99. doi: 10.1016/j.fertnstert.2018.01.038 29778388

[197] Helvaci N Karabulut E Demir AU Yildiz BO. Polycystic ovary syndrome and the risk of obstructive sleep apnea: a meta-analysis and review of the literature. Endocrine connections. (2017) 6:437–45. doi: 10.1530/EC-17-0129 PMC5574283 28739562

[198] Lin AW Bergomi EJ Dollahite JS Sobal J Hoeger KM Lujan ME. Trust in physicians and medical experience beliefs differ between women with and without polycystic ovary syndrome. J Endocrine Society. (2018) 2:1001–9. doi: 10.1210/js.2018-00181 PMC6101505 30140785

[199] Soucie K Samardzic T Schramer K Ly C Katzman R. The diagnostic experiences of women with polycystic ovary syndrome (PCOS) in ontario, Canada. Qual Health Res. (2021) 31:523–34. doi: 10.1177/1049732320971235 33213256

[200] Park S Lim SY Kim JY Park H Lim JS Bae S. Clinical and virological characteristics of severe acute respiratory syndrome coronavirus 2 (SARS-coV-2) B.1.617.2 (Delta) variant: A prospective cohort study. Clin Infect diseases: an Off Publ Infect Dis Soc America. (2022) 75:e27–34. doi: 10.1093/cid/ciac239 PMC9047158 35362530

[201] Hoang VT Colson P Levasseur A Delerce J Lagier JC Parola P. Clinical outcomes in patients infected with different SARS-CoV-2 variants at one hospital during three phases of the COVID-19 epidemic in Marseille, France. Infection Genet evolution: J Mol Epidemiol evolutionary Genet Infect diseases. (2021) 95:105092. doi: 10.1016/j.meegid.2021.105092 PMC8462069 34571275

[202] Fernández-de-Las-Peñas C Cancela-Cilleruelo I Rodríguez-Jiménez J Gómez-Mayordomo V Pellicer-Valero OJ Martín-Guerrero JD. Associated-onset symptoms and post-covid-19 symptoms in hospitalized COVID-19 survivors infected with wuhan, alpha or delta SARS-coV-2 variant. Pathog (Basel Switzerland). (2022) 11(7):725. doi: 10.3390/pathogens11070725 PMC9320021 35889971

[203] Menges D Ballouz T Anagnostopoulos A Aschmann HE Domenghino A Fehr JS. Burden of post-covid-19 syndrome and implications for healthcare service planning: A population-based cohort study. PloS One. (2021) 16:e0254523. doi: 10.1371/journal.pone.0254523 34252157 PMC8274847

[204] Kannan SR Spratt AN Cohen AR Naqvi SH Chand HS Quinn TP. Evolutionary analysis of the Delta and Delta Plus variants of the SARS-CoV-2 viruses. J autoimmunity. (2021) 124:102715. doi: 10.1016/j.jaut.2021.102715 34399188 PMC8354793

[205] Chen J Wang R Wang M Wei GW. Mutations strengthened SARS-coV-2 infectivity. J Mol Biol. (2020) 432:5212–26. doi: 10.1016/j.jmb.2020.07.009 PMC7375973 32710986

[206] Ningombam SS Kumar R Tanwar P. Mutant strains of SARS-CoV-2 are more prone to infect obese patient: a review. Wiener klinische Wochenschrift. (2021) 133:383–92. doi: 10.1007/s00508-021-01819-w PMC7887545 33595720

[207] Alefishat E Jelinek HF Mousa M Tay GK Alsafar HS. Immune response to SARS-CoV-2 variants: A focus on severity, susceptibility, and pre-existing immunity. J infection Public Health. (2022) 15:277–88. doi: 10.1016/j.jiph.2022.01.007 PMC8757655 35074728

[208] Cui J Li F Shi ZL. Origin and evolution of pathogenic coronaviruses. Nat Rev Microbiol. (2019) 17:181–92. doi: 10.1038/s41579-018-0118-9 PMC7097006 30531947

[209] Mongioì LM Barbagallo F Condorelli RA Cannarella R Aversa A La Vignera S. Possible long-term endocrine-metabolic complications in COVID-19: lesson from the SARS model. Endocrine. (2020) 68:467–70. doi: 10.1007/s12020-020-02349-7 PMC7266418 32488837

[210] Teede HJ Misso ML Costello MF Dokras A Laven J Moran L. Recommendations from the international evidence-based guideline for the assessment and management of polycystic ovary syndrome. Fertility sterility. (2018) 110:364–79. doi: 10.1016/j.fertnstert.2018.05.004 PMC6939856 30033227

[211] Tay MZ Poh CM Rénia L MacAry PA Ng LFP. The trinity of COVID-19: immunity, inflammation and intervention. Nat Rev Immunol. (2020) 20:363–74. doi: 10.1038/s41577-020-0311-8 PMC7187672 32346093

[212] Nalbandian A Sehgal K Gupta A Madhavan MV McGroder C Stevens JS. Post-acute COVID-19 syndrome. Nat Med. (2021) 27:601–15. doi: 10.1038/s41591-021-01283-z PMC8893149 33753937

[213] Wu Q. Understanding the burden of post-covid-19 condition. BMJ (Clinical Res ed). (2023) 381:932. doi: 10.1136/bmj.p932 37257892

[214] Teoh WS Ramu D Indran IR Chua MWJ Thu WPP Yong EL. Diagnosis and management of polycystic ovary syndrome: Perspectives of clinicians in Singapore. Ann Acad Medicine Singapore. (2022) 51:204–12. doi: 10.47102/annals-acadmedsg. 35506403

